# What Shapes the Genetic Diversity of the *Alnus cordata* Species Across Its Italian Native Range? Informing Conservation Strategies

**DOI:** 10.1002/ece3.72018

**Published:** 2025-08-31

**Authors:** Paola Pollegioni, Alexis Marchesini, Muriel Gaudet, Francesca Chiocchini, Flavio Monti, Luca Leonardi, Marcello Cherubini, Claudia Mattioni

**Affiliations:** ^1^ Research Institute on Terrestrial Ecosystems, National Research Council Porano Italy; ^2^ National Biodiversity Future Center Palermo Italy; ^3^ Research Institute on Terrestrial Ecosystems, National Research Council Lecce Italy

**Keywords:** alders, conservation strategies, genetic diversity, habitat suitability modeling, hybridization, microsatellites

## Abstract

*Alnus cordata* is an endemic tree species native to the Southern Italian Apennines and north‐eastern Corsica, renowned for its ecological significance. Climate change projections for the Mediterranean basin indicate range shifts and increased fragmentation for many forest trees, including *A. cordata* . Hybridization with the sympatric *A. glutinosa* in the central part of its Italian native range may also influence the genetic structure and conservation priorities for *A. cordata* . A comprehensive conservation strategy is needed to preserve its genetic resources in Italy. We analyzed the genetic diversity, population structure, and extent of hybridization with *A. glutinosa* in 23 *A. cordata* forest stands across its native Italian range using nuclear microsatellites. Habitat suitability was modeled under current and future climate scenarios using an ensemble forecasting approach. Conservation prioritization was guided by a genetically informed Reserve Selection analysis in DIVA‐GIS to identify areas of high conservation value and address gaps in genetic resource representation. Italian alder populations exhibit low genetic diversity, which decreases towards the southern latitudinal margins of the peninsula. Hybridization and introgression with *Alnus glutinosa* have a geographically localized impact on the genetic variation within *A. cordata* populations. Local increases in private allelic richness do not alter the spatial genetic structure of *A. cordata* , but they help mitigate the risk of severe genetic erosion. A significant proportion of the species' genetic diversity is effectively preserved through in situ conservation. Model projections under future climate scenarios indicate a substantial decline in habitat suitability for *A. cordata* stands with high priority for in situ conservation. This highlights the need for complementary strategies, including ex situ conservation measures. Our study highlights the importance of integrating genetic analyses, habitat suitability modeling, and spatial prioritization techniques for effective conservation planning of *A. cordata* in the face of climate change across the Mediterranean.

## Graphical abstract

*Alnus cordata* is an endemic tree species native to the Southern Italian Apennines and north‐eastern Corsica, renowned for its ecological significance. We analyzed the genetic diversity, population structure, and extent of hybridization with *A. glutinosa* in 23 *A. cordata* forest stands across its native Italian range. Conservation prioritization was guided by a genetically informed Reserve Selection analysis in DIVA‐GIS to identify areas of high conservation value and address gaps in genetic resource representation.

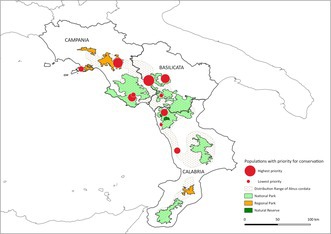

## 1 Introduction

Alnus Mill. (Betulaceae), commonly known as alders, comprises a genus of approximately 35 deciduous, wind‐pollinated tree and shrub species (Vít et al. 2017). Alders are pioneer species in riparian and mountainous habitats of temperate regions, also due to their nitrogen‐fixing association with *Frankia* sp. (Buchbauerová et al. 2024). Their rapid growth, nitrogen fixation, and coppicing potential make them valuable for nature‐based solutions (NbS; Dhyani et al. 2020) such as afforestation, reforestation, and land reclamation (Innangi et al. 2017; Manetti et al. 2022), urban tree planting (Battisti et al. 2023), biomass energy production (Daugaviete et al. 2022), and mixed‐species plantations (Danise et al. 2021). Alders also contribute to flood control, riverbank stabilization, slope restoration, and ecosystem rehabilitation (Kastridis et al. 2021). One notable species, *Alnus cordata* (Loisel.) Duby, known as “Italian alder” is a Tyrrhenian endemic monoecious species, primarily outcrossing (Heuvel 2011), native to the Southern Italian Apennines and northeastern Corsica (Caudullo and Mauri 2016). In its native range, *A. cordata* grows in the sub‐mountain and mountain belt, preferring moist soil and climates with an annual precipitation of at least 1000 mm per year. However, it exhibits more drought tolerance and less dependence on riparian habitats compared to other alder species (Caudullo and Mauri 2016). Under optimal conditions, it forms pure stands alongside *Quercus cerris* and *Fagus sylvatica* forests (Ducci and Tani 2009). Due to its nitrogen fixation capacity, *A. cordata* tends to colonize *Pinus nigra* plantations, abandoned *Castanea sativa* orchards, and even eroded and exposed soils following wildfires or landslides (Ducci and Tani 2009). Despite its limited natural distribution and the current threats primarily related to forest management practices (e.g., heavy unauthorized grazing, and reduction of mixed forest management), *A. cordata* is not listed as an endangered species according to IUCN (Shaw et al. 2017). However, under future climate change scenarios predicted in the Mediterranean Basin, a biodiversity hotspot (Médail and Quezel 1997), increased temperature and altered water availability may lead to a shift in Italian alder's spatial distribution and promote marked fragmentation of its natural stands (Attorre et al. 2011). Consequently, genetic drift and inbreeding events could have detrimental effects on the viability of *A. cordata* populations (Brook et al. 2002; Reed and Frankham 2003).

In this context, studying the population genetics of *A. cordata* is crucial for evaluating the species' evolutionary potential to adapt to a changing environment. Similar to several forest trees, monitoring levels of genetic diversity within and among *A. cordata* populations can provide vital information for identifying genetic diversity hotspots (Souto et al. 2015). Conservation genetic information can ultimately enhance the effectiveness of in situ conservation strategies by guiding the implementation of a network of forest stands identified as Genetic Conservation Units and selecting plant material for ex situ conservation measures (Koskela et al. 2013; Kelleher et al. 2015). Genetic monitoring has been already performed in several alder species ranging from the widely distributed Holarctic species *Alnus alnobetula* (Ehrh.) K. Koch s.l. (Hantemirova and Marchuk 2021) and *Alnus incana* (L.) Moench (Mandák et al. 2016), to American species *Alnus rubra* Bong. (Xie et al. 2002) and *Alnus maritima* (Marsh.) Muhl. ex Nutt (Jones and Gibson 2011). Studies on the postglacial history reconstruction of the European alder species *Alnus glutinosa* (L.) Gaertn and its adaptive genomic variation have been conducted at both continental (Havrdová et al. 2015) and national scales (Verbylaitė et al. 2023; Mingeot et al. 2016). Plastid phylogenetic analysis of *A. cordata* and *A. glutinosa* suggests paleo‐introgression events after the last interglacial (< 100,000 years ago) (King and Ferris 2000). Although rare F2 hybrids and backcrosses were found, recent studies confirm low levels of hybridization and introgression in sympatric populations of Southern Italy (Villani et al. 2021; Gryta et al. 2017). To date, no other genetic surveys have been carried out for this Mediterranean endemic tree, and its genetic diversity throughout the Italian distributional range remains largely unknown.

The aim of this study was to delineate best practices for the long‐term preservation of *A. cordata* genetic diversity, incorporating both in situ and ex situ strategies across Southern Italy in response to ongoing global environmental changes. To achieve this, we primarily evaluated the genetic population structure and within‐population diversity of *Alnus cordata* across the Italian native range. However, the documented hybridization events in the species' central distribution core (Villani et al. 2021) provided a unique opportunity to quantify the extent of hybridization with *A. glutinosa* in *A. cordata* populations and assess its potential impact on their genetic diversity and structure. Finally, we applied the gap analysis, a well‐established conservation technique (Maxted et al. 2008), to (1) identify current geographic sites with high priority for in situ conservation of *A. cordata* genetic resources, (2) detect elements of genetic diversity underrepresented in the selected protected areas (gap elements) and (3) predict how sites with high priority for in situ conservation would be affected under different climate change scenarios in the future.

## 2 Methods

### 2.1 Plant Material

**FIGURE 1:**
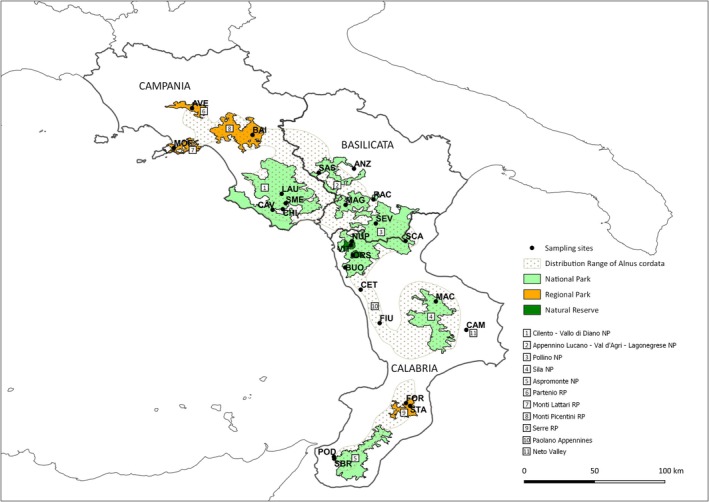
Location of 25 populations of *Alnus* sp. collected across its Italian native range according to Euforgen (Available online http://www.euforgen.org/species/, accessed on 31 December 2023) in the Paolano Ridges and nine protected macro areas: Regional Park of Partenio Mountains, Regional Park of Lattari Mountains, Regional Park of Picentini Mountains, National Park of Cilento—Vallo di Diano Mountains sited in Campania region, National Park Appennino Lucano Val d'Agri Lagonegrese Mountains located in Basilicata region, National Park of Pollino Mountains connecting the Tyrrhenian Sea cost of Calabria to the inland of Basilicata, and three Parks distributed across Calabria region, National Park of Sila Mountains, Regional Park of Serre, and National Park of Aspromonte Mountains. Each population where the sampling was carried out is indicated by a black dot and a three‐letter code. More details on *A. cordata* populations are shown in Table 1.

According to the species distribution range (Euforgen map, Available online http://www.euforgen.org/species/, accessed on 31 December 2023) and the census conducted by the Regional Forestry Agency, 25 natural populations of alder trees, *Alnus glutinosa* and *A. cordata* , were collected across the Apennine Mountains spanning three regions of Southern Italy—Campania, Basilicata, and Calabria—through two sampling campaigns performed in April 2012 (Villani et al. 2021) and May 2022 (Figure 1). In agreement with the species occurrence points of *A. cordata* (GBIF.org [6 November 2024] GBIF Occurrence Download https://doi.org/10.15468/dl.nazq94) and *A. glutinosa* (GBIF.org [14 July 2025] GBIF Occurrence Download https://doi.org/10.15468/dl.cg4ejg), 17 to 25 trees per site were sampled and georeferenced for a total of 518 individuals (Table 1). The sampling strategy included three alder natural populations from the Paolano Ridges and the Neto Valley in the coastal zones of the Region Calabria, and 22 alder natural populations sampled in nine protected macro areas: Regional Park (RP) of Partenio Mountains (1), RP of Lattari Mountains (1), RP of Picentini Mountains (1), National Park (NP) of Cilento—Vallo di Diano Mountains (4) in Campania, NP Appennino Lucano Val d'Agri Lagonegrese Mountains (4) located in Basilicata, NP of Pollino Mountains (6) covering an area of almost 200,000 ha and connecting the Tyrrhenian Sea coast of Calabria to the inland of Basilicata, and three Parks distributed across the Calabria region, NP of Sila Mountains (1), RP of Serre (2), and NP of Aspromonte Mountains (2) (Figure 1). It is worth noting that only a sparse number of *A. cordata* trees were located and genotyped at the STA stand owing to their scattered and discontinuous presence in the uppermost and coldest regions of the RP of Serre. During the sampling campaign, a notable presence of *A. glutinosa* was identified. Despite exhaustive efforts to survey the STA area, no additional individuals (5) of the *A. cordata* species were found. Potential evidence for hybridization between *A. cordata* and its closely related species *A. glutinosa* has been reported in Corsica Island (King and Ferris 2000) and Southern Italy (Villani et al. 2021). The preliminary taxonomic classification of each sampled tree as *A. cordata* and *A. glutinosa* was performed in the field according to the morphology of leaves and bark, in line with the methodology described by Villani et al. (2021). A total of seven admixed alder populations, two pure stands of *A. glutinosa* , and 16 pure stands of *A. cordata* were identified, with a total of 422 *Alnus cordata* and 96 *A. glutinosa* trees (Table 1). Leaves from each tree were collected and stored at −80°C.

**TABLE 1:** Geographic and sampling information of seven admixed alder populations (cord/glut), two pure populations of *A. glutinosa* (glut) and 16 pure populations of *A. cordata* (cord): Code (Popcode), Population site (Pop_name), region (Region), geographic coordinates in decimal degrees, latitude (Lat.) and longitude (Long.), elevation (m) above sea level (Elev.), and number of individuals sampled (*N*. individuals).

Species	Popcode	Pop_name	Region	Long.	Lat.	Elev.	Description	*N*. individuals		
								cord	glut	Total
cord	AVE	Avella	Campania	14.6421	40.9797	773.00	Regional Park of Partenio	21		21
cord	MOF	Monte Faito	Campania	14.4951	40.6580	1211.00	Regional Park of Lattari Mountains	18		18
cord	BAI	Bagnoli Irpino	Campania	15.1301	40.7640	1259.16	Regional Park of Picentini Mountains	20		20
cord	LAU	Laurino	Campania	15.3657	40.2883	954.38	National Park of Cilento—Vallo di Diano	24		24
cord/glut	SME	San Menale	Campania	15.3984	40.2121	552.16	National Park of Cilento—Vallo di Diano	16	9	25
cord	CAV	Cavallara	Campania	15.2922	40.1595	686.89	National Park of Cilento—Vallo di Diano	20		20
cord	CHI	Chiaie	Campania	15.3747	40.1644	698.20	National Park of Cilento—Vallo di Diano	20		20
cord	RAC	Torrente Nocito	Basilicata	16.1074	40.2431	386.17	National Park Appennino Lucano Val d'Agri Lagonegrese	18		18
cord	MAG	Torrente Maglie	Basilicata	15.8821	40.1990	744.30	National Park Appennino Lucano Val d'Agri Lagonegrese	20		20
cord/glut	SAS	Sasso di Castalda	Basilicata	15.6645	40.4587	744.33	National Park Appennino Lucano Val d'Agri Lagonegrese	16	9	25
cord/glut	ANZ	Anzi	Basilicata	15.9498	40.4911	583.88	National Park Appennino Lucano Val d'Agri Lagonegrese	14	3	17
cord/glut	SEV	San Severino	Basilicata	16.1260	40.0489	624.05	National Park of Pollino	13	6	19
cord	NUP	Nuppolara	Calabria	15.9337	39.9053	396.61	National Park of Pollino	18		18
cord/glut	BUO	Buonvicino	Calabria	15.8789	39.6933	199.96	National Park of Pollino	17	8	25
cord/glut	VIT	Vitimoso	Calabria	15.9246	39.8734	173.04	National Park of Pollino	16	9	25
cord	ORS	Orsomarso	Calabria	15.9409	39.7928	212.26	National Park of Pollino	19		19
cord	SCA	Scaragiani	Calabria	16.3668	39.9073	1166.22	National Park of Pollino	19		19
cord	CET	Cetraro	Calabria	16.0039	39.5147	620.80	Paolano Appennines‐Cetraro	20		20
cord	FIU	Fiumefreddo	Calabria	16.1570	39.2444	1015.20	Paolano Appennines‐Mendicino	25		25
cord	MAC	Macrocioli	Calabria	16.6107	39.4194	1275.68	National Park of Sila	24		24
glut	CAM	Santa Severina	Calabria	16.8576	39.1889	100.00	Neto Valley		17	17
cord	FOR	Forgevecchie	Calabria	16.3697	38.5985	950.68	RPSerre	19		19
cord/glut	STA	Stafferino	Calabria	16.4028	38.5759	1023.27	RPSerre	5	17	22
cord	SBR	San Bruno	Calabria	15.7903	38.1499	1037.70	National Park of Aspromonte	20		20
glut	POD	San Bruno	Calabria	15.7903	38.1646	616	National Park of Aspromonte		18	18
	Total							422	96	518

*Note:* The preliminary taxonomic classification of each alder tree was performed in the field according to morphology of leaves and bark, in line with the methodology described by Villani et al. (2021).

### 2.2 DNA Isolation, SSR Amplification and Genotyping of *Alnus* Trees

Dehydrated leaf tissue (60 mg) from each sample was homogenized in a 2‐ml microcentrifuge tube containing a 5‐mm steel bead cooled with liquid nitrogen using Mixer Mill 300 (Qiagen, Hilden, Germany). Genomic DNA was extracted and purified using a DNeasy 96 Plant Kit (Qiagen) and stored at −20°C. In this study, all *Alnus* sp. samples were genotyped using 10 nSSR loci (A2, A22, A35, A37, A38, AG10, AG13, alma1, alma11, and alng4), mapped in seven of the 14 total chromosomes of the *Alnus glutinosa* genome assembly dhAlnGlut1, and already selected and used for the genetic characterization of *Alnus* tree species across Europe (Mandák et al. 2016; Havrdová et al. 2015; Villani et al. 2021) (for a complete description of the SSR loci, see Appendix S1 and Tables S1 and S2). PCR amplification and the visualization of amplified SSR alleles for each sample were carried out as described by Villani et al. (2021). The allele size scoring was performed using GeneMapper version 4 (Applied Biosystems, Foster City, CA, USA), using eight *Alnus cordata* genotypes already characterized by SSR markers (Villani et al. 2021) as standards across multiple plates.

### 2.3 Identification and Classification of Alder Tree Hybrids

Due to the complex hybridization and introgression processes detected among *Alnus* species (Jurkšienė et al. 2021), we performed the hybrid detection of alders using a dataset that combined *A. cordata* and *A. glutinosa* microsatellite genotypes prior to further intraspecific analyses. First, to display the genetic distances between 518 alder trees, a Principal Coordinate Analysis (PCoA) based on the pairwise individual genetic distance (GD) matrix (Smouse and Peakall 1999) was performed using GenAlEx software. Subsequently, the model‐based Bayesian statistical approach implemented in NEWHYBRIDS software version 1.1 (Anderson and Thompson 2002) was applied following the procedure described by Pollegioni et al. (2013). This method provides the posterior probability (*Q*i) of each alder individual belonging to six default genotype frequency classes: (QI) Purebred_ *A. cordata* , (QII) Purebred_ *A. glutinosa* , (QIII) F1 hybrid, (QIV) F2 hybrid, (QV) BC_ *A. cordata* (backcross towards *A. cordata* ), and (QVI) BC_ *A. glutinosa* (backcross towards *A. glutinosa* ). Six independent runs were carried out with a burn‐in period of 20,000 generations and 200,000 MCMC replicates, with no previous population information and Jeffery's like prior parameters. In this study, a strict posterior probability of 90% was considered a statistical threshold for assignment analysis. To perform a more detailed analysis of admixture proportions and confirm the previous species assignment, a Bayesian model‐based clustering method implemented in STRUCTURE software 2.3.3 (Pritchard et al. 2000) was used to assign alder genotypes to *A. cordata* and *A. glutinosa* species and identify putative hybrids. This method attempts to assign individuals to *K*‐genetic clusters to minimize within‐group linkage disequilibrium and deviation from Hardy–Weinberg equilibrium, computing the admixture coefficient or proportion of membership (*Q* value) for each genotype. STRUCTURE analysis was performed using the admixture model on the whole data set with no previous population information, the correlated allele frequencies between populations, and a burn‐in period of 10,000 steps followed by 10^5^ MCMC replicates options. We conducted six independent runs at each *K* from 1 to 10 to determine the most likely number of clusters according to post hoc statistics of Evanno et al. (2005) implemented in StructureSelector software (Li and Liu 2018). Based on the initial results, we assumed *K* = 2 as the most likely number of clusters in agreement with the presence of two species (*Q*_1_ *A. cordata*_ = *A. cordata* , *Q*_2_ *A. glutinosa*_ = *A. glutinosa* ). Therefore, as suggested by Pollegioni et al. (2013), we classified individuals with admixture coefficient *Q*_1_ *A. cordata*_ ≥ 0.9 as *A. cordata* , *Q*_2_ *A. glutinosa*_ ≥ 0.9 as *A. glutinosa* , and 0.11 ≤ *Q*_1_ *A. cordata*_ ≤ 0.89 as hybrids. The six runs from the most probable number of clusters *K* = 2 or for the six‐default genotype frequency (QI–QVI) classes were averaged and graphically displayed as reported by Pollegioni et al. (2013). Due to a few slightly contrasting results provided by STRUCTURE and NewHybrids analyses, we performed a maximum‐likelihood estimation of the hybrid index (HD), measured as the proportion of alleles with *A. glutinosa* ancestry, by the INTROGRESS R package (Gompert and Alex Buerkle 2010) for all individuals from seven sites of sympatry. We defined a priori parent species 1 ( *A. cordata* ) as any tree alder from pure *A. cordata* stands showing *Q*_1_ *A. cordata*_ ≥ 0.9 and a priori parent species 2 ( *A. glutinosa* ) as any tree alder from pure *A. glutinosa* stands showing *Q*_2_ *A. glutinosa*_ ≥ 0.9. As described by Larson et al. (2013), individuals were generally classed as F1 hybrids (HD = 0.5, interspecific heterozygosity ≥ 85%), multi‐generation hybrids (HD = 0.25–0.75, interspecific heterozygosity < 85%), back‐crossed to *A. cordata* (HD = 0.10–0.25, interspecific heterozygosity < 85%) or backcrossed into *A. glutinosa* (HD = 0.75–0.90, interspecific heterozygosity < 85%). Individuals with hybrid indices < 0.1 or > 0.9 represented pure *A. cordata* and pure *A. glutinosa* , respectively.

### 2.4 Genetic Diversity of the *A. cordata* Populations

After excluding interspecific alder hybrids from our dataset, we assessed the genetic diversity of each *A. cordata* population using nine polymorphic nSSRs. Genetic diversity was evaluated based on the mean number of alleles per locus (*A*), observed heterozygosity (*H*_o_), expected heterozygosity (*H*_E_), and unbiased expected heterozygosity corrected for sample size (UH_e_). All analyses were performed with GenAlEx version 6.3 (Peakall and Smouse 2006). To account for differences in sample size, allelic richness (*R*_s_) and private allele richness (PAr) were computed by the rarefaction method with HP‐Rare software (Kalinowski 2004). Considering the low number of alder trees sampled at the STA site, *R*_s_ and PAr tables were normalized to the second lowest number of sampled trees per population (10 individuals). Following the procedure of Pollegioni et al. (2014), the Inverse Distance Weighted (IDW) interpolation function implemented in the Geographic Information System software QGIS 3.18 (Open‐Source Geospatial Foundation Project, Available online: http://www.qgis.org) was used to display the geographic patterns of *R*_s_, UH_e_, and PAr computed for 22 Italian alder populations. The within‐population inbreeding coefficient *F*_IS_ (Weir and Cockerham 1984) was estimated for each population using hierarchical locus‐by‐locus AMOVA as implemented in Arlequin 3.11 software (Excoffier et al. 2005). The statistical significance of *F*_IS_ was tested using a nonparametric approach with 1000 permutations. Finally, evidence of very recent population size decrease was investigated using the program BOTTLENECK 1.2.02 (Piry et al. 1999). As described by Pollegioni et al. (2014), significance was assessed using the “Wilcoxon's signed rank” test and TPM with 70% Stepwise Mutations Model (SMM) and 30% multistep mutations. For each mutational model, 10,000 replicates were performed. Two additional tests were used to identify bottleneck signatures from larger time scales: shifted allele distribution analysis (Luikart et al. 1998) and the M‐ratio test (Garza and Williamson 2001) implemented in BOTTLENECK and Arlequin software, respectively.

### 2.5 Genetic Structure of the *A. cordata* Populations

Three complementary statistical approaches were used to detect and quantify the spatial genetic structure of Italian alder populations across its native range. First, the distribution of the genetic diversity within, among alder populations, and among NP/RPs was investigated with hierarchical locus‐by‐locus AMOVA analysis using Arlequin software. *F*_ST_ coefficients following Weir and Cockerham (1984) were also calculated between pairs of sampling sites. The significance of *F*_ST_ was estimated with 10,000 random permutations. To display the genetic differentiation between *A. cordata* populations, Principal Coordinate Analysis (PCoA) and a heatmap analysis based on the pairwise *F*_ST_ matrix were performed using the *heatmap.2* function implemented in the R package “*gplots*” (Warnes et al. 2022) and GenAlEx software. Second, a Bayesian clustering approach implemented in STRUCTURE 2.3.3 software (Pritchard et al. 2000) was applied. STRUCTURE analysis was performed and displayed as described above. The range of the possible number of clusters (*K*) tested was from 1 to 24. Finally, to confirm the genetic repartition of alder populations inferred by STRUCTURE, a UPGMA tree was constructed based on Nei's (1972) genetic distance (DA) using POPTREE2 software (Takezaki et al. 2010). The stability of the cluster was tested through 1000 bootstraps resamples.

### 2.6 Correlation Between Genetic and Landscape Data

To determine whether within‐ and between‐population genetic variation of *A. cordata* correlated with geographic gradients, we conducted two main analyses: (1) Pearson correlation analysis; and (2) a simple Linear Modeling (LM) analysis of *R*_s_, UH_e_, and PAr *against* latitude, longitude, and elevation of sampled sites. Additionally, we assessed the potential impact of interspecific hybridization and/or introgression between *A. cordata* and *A. glutinosa* on the genetic diversity of *A. cordata* populations throughout the Southern Apennines. We included both the mean HD and the estimated mean *Q*_2_*A. glutinosa*_ inferred for each *A. cordata* population into our correlation and model analysis. After excluding variables with a Pearson's coefficient |*r*| ≥ 0.80, we developed comprehensive models that incorporated latitude, longitude, and elevation as geographic predictors and *Q*_2_*A. glutinosa*_ as a predictor of introgression between *A. cordata* and *A. glutinosa* . Following a conservative approach (Richards et al. 2011), we did not select models with Akaike's information criterion corrected for small sample sizes (AICc) ≥ 2 in respect to the best model, as well as models with an AICc value greater than that of any simpler alternative. All the analyses were performed in R version 4.0.3 (R Core Team 2020), through the packages *MuMIn* (for model selection; Bartoń 2013) and *lme4* (for LMs; Bates et al. 2015).

Isolation by distance (IBD) and gene flow among *A. cordata* populations were analyzed using a Mantel test (Mantel 1967) and Mantel Bearing correlogram, respectively, using the software PASSaGE (Rosenberg and Anderson 2011). Isolation by distance was evaluated by regressing Slatkin's linearized [*F*_ST_/(1 − *F*_ST_)] values against spherical geographic distances (km). Mantel Bearing correlogram was built considering seven geographic distance classes; the upper limits of these distance classes were 33.021, 53.217, 65.249, 76.886, 101.289, 134.370, and 159.331 km. This directional spatial autocorrelation method consists of setting “distance/direction classes” that not only contain information about the distance between two points but also the direction. Significance was assessed via 1000 permutations.

### 2.7 Prediction of the Priority Areas for Conservation

The DIVA‐GIS software (www.diva‐gis.org) was used to define the areas with high priority for conservation of *A. cordata* species across its Italian native range. We used the Reserve Selection analysis that is based on an optimization algorithm developed by Rebelo and Siegfried (1992). A point‐to‐grid analysis was applied, selecting the “Complementary” option, and giving equal weight to each allele. As reported in Mattioni et al. (2017), this method allows the selection of conservation priority areas not only based on their allelic richness but also on differences/complementarity in allelic composition, by identifying the minimum number of sites needed to conserve 100% of genetic diversity estimated by nuclear markers.

### 2.8 Species Distribution Modeling of *A. cordata*

In this study, species occurrence points of *A. cordata* were collected from Italy and Corsica using (a) the Global Biodiversity Information Facility database (GBIF.org [6 November 2024] GBIF Occurrence Download https://doi.org/10.15468/dl.nazq94), (b) the EU‐Forest dataset (Mauri et al. 2017), and through two field surveys performed in 2012 and 2022 across Southern Italy. Besides our own 417 locations, another 2533 records were gathered for a total of 2950 data points. Duplicated records falling into the same pixel of the environmental variables and points occurring in the sea were excluded. As a result, the number of georeferenced records was reduced to 1877. We calibrated SDM considering 19 bioclimatic layers with 30 arc sec grid cells downloaded from the Worldclim website (Fick and Hijmans 2017). To avoid multicollinearity and prevent overfitting, variance inflation factors (VIFs) and Pearson correlation coefficients were calculated among bioclimatic variables using the *usdm* R package (Naimi et al. 2014). Bio‐variables having a high VIF > 7 and a Pearson's coefficient |*r*| ≥ 0.85 were excluded from further analysis. Among two highly correlated variables, one was chosen for its biological relevance for the species. Indeed, eight variables were included: Isothermality (BIO3), Temperature Seasonality (BIO4), Mean Temperature of Wettest Quarter (BIO8), Mean Temperature of Driest Quarter (BIO9), Precipitation of Driest Quarter (BIO17), Precipitation of Warmest Quarter (BIO18) and Precipitation of Coldest Quarter (BIO19). As described in Di Febbraro et al. (2019), SDM was calibrated through an ensemble forecasting approach implemented in the *biomod2* R package (Thuiller et al. 2009). We selected five algorithms repeatedly used in several plant species (Lozano et al. 2023) including alders (Rana et al. 2018; Mandák et al. 2016): generalized linear models (GLM), generalized additive models (GAM), generalized boosted models (GBM), random forests (RF) and maximum entropy models (MAXENT). A set of 10,000 background points was generated in an area encompassing the whole Italian Peninsula and Corsica Island. Of the records, 80% were randomly selected for modeling, and another 20% were used for validation. The resulting model projections were the medians from two replicates. The predictive performance of each model was assessed by measuring the area under the receiver operating characteristic curve (AUC) and the true skill statistic (TSS). To avoid using poorly calibrated models, only projections from models with AUC ≥ 0.6 were considered in further analyses. Model averaging was performed by weighting the individual model projections by their AUC scores and averaging the result (Di Febbraro et al. 2019). We calculated the relative importance of variables from the ensemble model using the functionality provided in the *biomod2* package. Predictions of the future bioclimatic variables were derived from the EC‐Earth‐Consortium EC‐Earth3‐Veg model (EC‐Earth Consortium 2019) provided by the Coupled Model Intercomparison Project Phase 6 (CMIP6) with Representative Concentration Pathway 4.5 and 8.5 scenarios (RCP) for the time windows of 2041–2060 (2050) and 2061–2080 (2070). We reclassified the ensembled output current and future projections into four classes of habitat suitability using TSS cutoff as a threshold value: not suitable (0–0.25), low suitability (0.25–0.40), moderate suitability (0.40–0.60), and high suitability (> 0.60). Finally, we displayed the geographic results under the 2050 and 2070 climate change scenarios with the current potential distribution by QGIS.

## 3 Results

### 3.1 Hybrid Classification of Alder Trees

**FIGURE 2:**
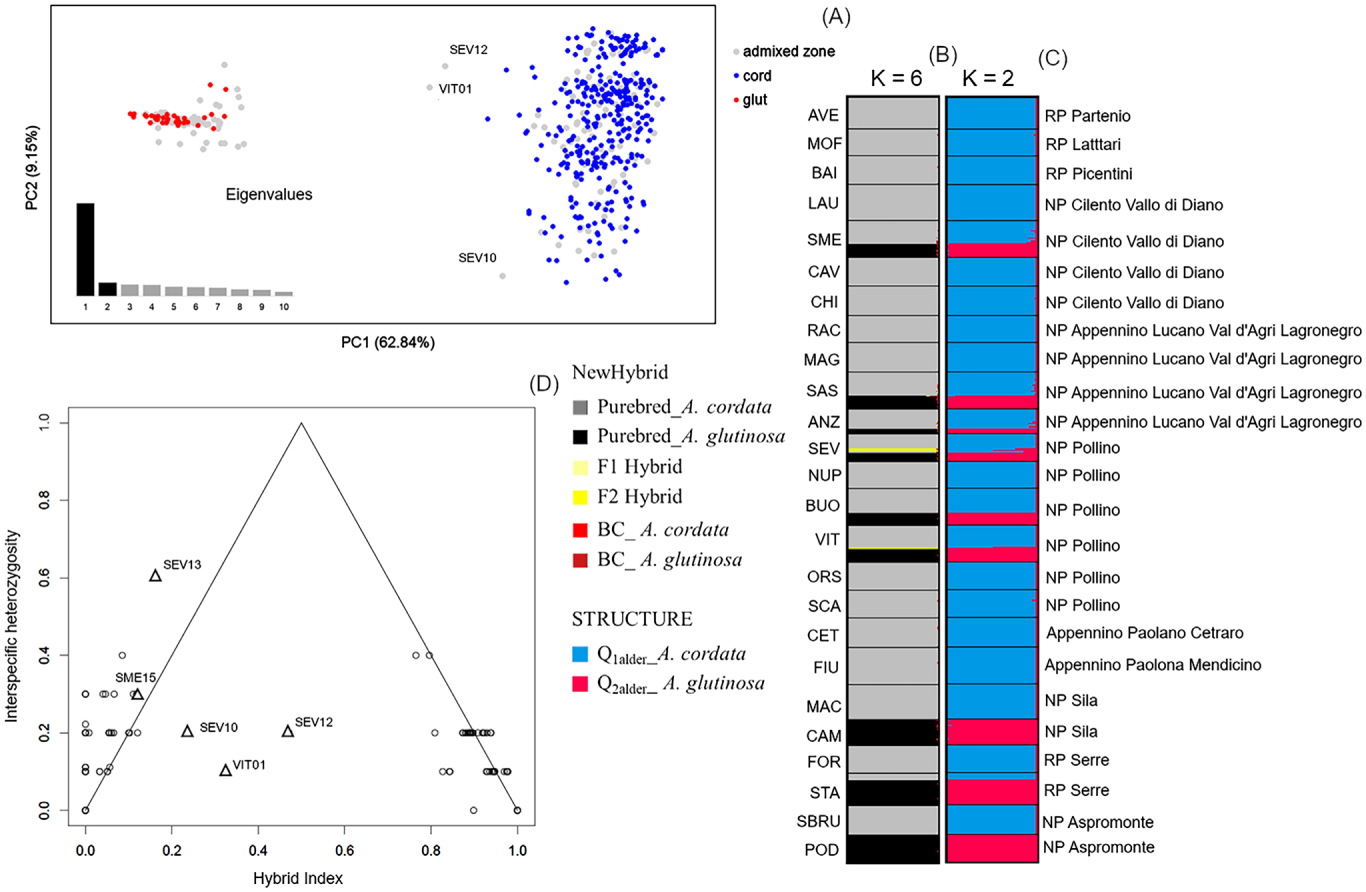
Hybridization between *A. cordata* and *A. glutinosa* considering the entire set of 518 alder trees collected across their native range in Italy. (A) Principal Coordinate Analysis of pairwise individual genetic distance (GD) values (Smouse and Peakall 1999) between 518 alder trees sampled in 16 pure stands of *A. cordata* (cord), two pure stands of *A. glutinosa* (glut) and seven admixed alder populations (unknown). (B) NEWHYBRIDS Bayesian assignments to six default genotype frequency classes: Purebred_ *A. cordata* , Purebred_ *A. glutinosa* , F1 hybrid, F2 hybrid, BC_ *A. cordata* (backcross towards *A. cordata* ), and BC_ *A. glutinosa* (backcross towards *A. glutinosa* ). (C) STRUCTURE Bayesian group assignments, in which the bar represents the individual's estimated membership percentage for *K* = 2 (*Q*_1_ *A. cordata*_ = *A. cordata* , *Q*_2_ *A. glutinosa*_ = *A. glutinosa* ). (D) Triangle plot summarizing interspecific heterozygosity vs. hybrid index, measured as the proportion of alleles with *A. glutinosa* ancestry for all individuals collected from seven sites of sympatry (dot plot), including five putative alder hybrids showing 0.11 ≤ *Q*_1_ *A. cordata*_ ≤ 0.89 (triangular plot).

All 10 SSR loci used in the present study were polymorphic across 25 sampled alder populations. A total of 125 alleles were detected in the 518 alder trees genotyped, with no evidence of null alleles (for a complete description of the SSR loci, see Appendix S1 and Tables S1 and S2). NEWHYBRIDS and PCoA analyses consistently identified three putative F2 hybrids: SEV_10, SEV_12, and VIT_01 (Figure 2A,B). Similarly, Bayesian admixture analysis using STRUCTURE effectively categorized individuals as *A. cordata* , *A. glutinosa* , or hybrids (Figure 2C). With *K* = 2 as the optimal number of genetic clusters (Figure S1) and a *Q*i threshold of ≥ 0.90, we confirmed two previously detected early hybrids, SEV_12 and VIT_01 (0.509 ≤ *Q*_1_ *A. cordata*_ ≤ 0.517), along with three putative later‐generation backcrosses to *A. cordata* —SME_15, SEV_10, and SEV_13 (0.764 ≤ *Q*_1_ *A. cordata*_ ≤ 0.889). These five trees, located in the NP of Pollino (SEV and VIT sites) and the NP of Cilento—Vallo di Diano (SME site), were clearly distinguishable from the 96 pure *A. glutinosa* individuals (*Q*_2_ *A. glutinosa*_ ≥ 0.917) and 417 pure *A. cordata* individuals (*Q*_1_ *A. cordata*_ ≥ 0.916) (Figure 2C). INTROGRESS analysis corroborated these findings but also suggested a broader pattern of introgression towards *A. cordata* or *A. glutinosa* compared to the Bayesian approach. Specifically, INTROGRESS identified seven backcrossed *A. cordata* individuals (HD = 0.10–0.19) across SME (4), SEV (2), and SAS (1) populations, three of which were already classified as backcrosses to *A. cordata* (Figure 2D). As expected, SEV_12 and VIT_01 exhibited multi‐generation hybrid scores of 0.471 and 0.325, respectively. Additionally, INTROGRESS classified 417 individuals as pure *A. cordata* , 77 as pure *A. glutinosa* , and 19 as backcrosses to *A. glutinosa* (HD scores = 0.765–0.899). Similar to the *A. cordata* backcrosses, these putative *A. glutinosa* backcrosses were distributed across the NP of Pollino [(2) SEV, (1) VIT, (1) BUO], the NP of Cilento—Vallo di Diano [(4) SME], and the NP of Appennino Lucano Val d'Agri Lagonegrese [(1) SAS, (2) ANZ], as well as the RP of Serre [(1) STA]. Finally, five individuals—VIT_01, SME_15, SEV_10, SEV_12, and SEV_13—were consistently identified as multi‐generation hybrids or backcrosses to *A. cordata* by at least two methods. As a result, they were excluded from the *A. cordata* dataset for subsequent genetic analyses.

### 3.2 Genetic Diversity of *A. cordata* Populations

**FIGURE 3:**
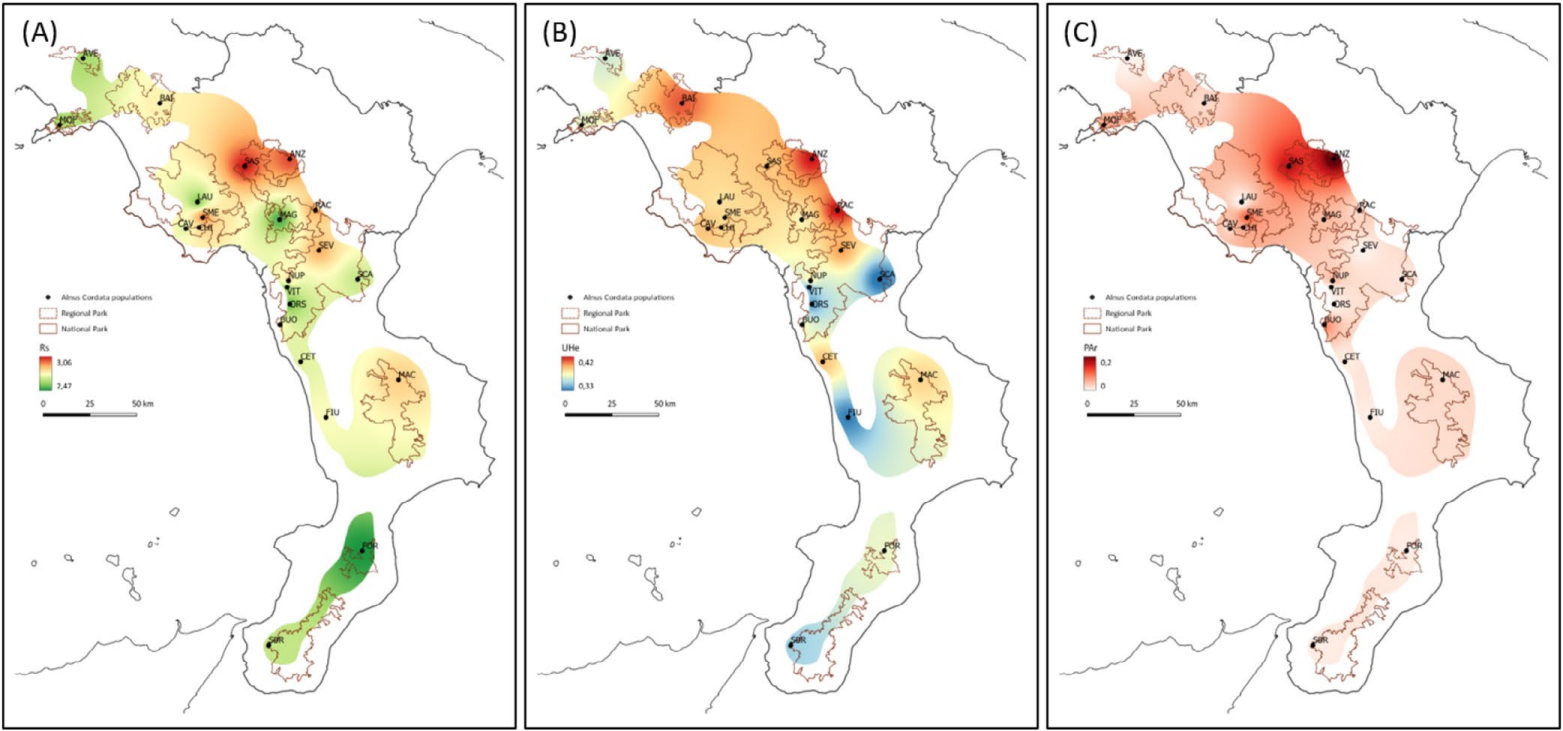
Genetic diversity of 22 populations of *A. cordata* across its Italian native range. Inverse Distance Weighted (IDW) interpolation of the allelic richness values (*R*_s_) (A), unbiased heterozygosity (UH_e_) (B) and private allelic richness (PAr) (C) values calculated for 22 Italian alder populations (black dots) collected in the Paolano Ridges and nine protected macro areas of Southern Italy using SSR markers. Considering the low number of *A. cordata* trees sampled at STA site, *R*_s_ and PAr were normalized to the second lowest number of sampled trees per population (10 individuals), thus STA is not displayed.

Genetic diversity estimates for 417 trees showed that *A. cordata* populations maintained relatively low levels of genetic diversity, with the highest mean number of alleles per locus at the FIU site (Table 2). The mean unbiased expected (UH_e_) and observed (*H*_o_) heterozygosity across all loci ranged from 0.213 (STA) to 0.425 (RAC) and from 0.200 (STA) to 0.450 (ANZ). Analogously, the allelic richness (*R*_s_) did not differ greatly among populations, varying from 2.470 (FOR) to 3.060 (SAS) with a minimum value (1.700) in the STA site (Table 2). However, two macro geographic areas showed the highest values of *R*_s_ and UH_e_: RP Monti Picentini (*R*_s_ = 3.140, UH_e_ = 0.402) including the BAI population sampled in the Campania region and RAC, MAG, SAS, and ANZ sites accounting for a large portion of NP of Appennino Lucano Val d'Agri Lagonegrese (*R*_s_ = 3.420, UH_e_ = 0.407) located in the Basilicata region (Table S3, Figure 3). Of 57 total alleles detected in *A. cordata* , 11 were unique to a single park; in particular, nine alleles were exclusive to NP Appennino Lucano Val d'Agri Lagonegrese (6) and NP Cilento—Vallo di Diano (3), consistent with their high rarified private allelic richness PAr (Table S3, Figure 3). Finally, more than half of the *A. cordata‐*private alleles detected across the RP Lattari Mountains, NP Appennino Lucano Val d'Agri Lagonegrese, NP Cilento—Vallo di Diano, and NP Pollino might have introgressed from *A. glutinosa* . Out of the 11 private alleles, seven (63.6%) in fact occur at low frequency in *A. cordata* , from one to three individuals, and are relatively common in *A. glutinosa* (range frequency from 3% to 11%). Following the exclusion of variables with a Pearson's coefficient |*r*| ≥ 0.80 (*R*_s_ vs. UH_e_ *r* = 0.844, *p* = < 0.001; *Q*_2_*A. glutinosa*_ vs. HD *r* = 0.883, *p* = < 0.001; see Figure S2), a positive correlation has been found between *R*_s_ and the latitude of the sampled sites (*r* = 0.436, *p* = 0.037) (Tables S4 and S5, Figure S2). Furthermore, a statistically significant positive trend has been detected between the mean *Q*_2_*A. glutinosa*_ inferred by STRUCTURE for each *A. cordata* population and the genetic diversity in terms of *R*_s_ (*r* = 0.462, *p* = 0.026) and PAr (*r* = 0.815, *p* < 0.001) (Tables S4 and S5, Figure S2).

**TABLE 2:** Genetic diversity of the *A. cordata* populations computed by SSR markers: Mean number of alleles per locus (*A*), effective number of alleles (*N*_e_), allelic richness (*R*_s_) and private allelic richness (PAr) standardized to the second lowest number of sampled trees per population (10 individuals), observed (*H*_o_), expected (*H*_E_), and unbiased expected heterozygosity (UH_e_), and inbreeding coefficient (*F*_IS_) are shown.

Population	*A*	*N*_e_	*R*_s_	*H*_o_	*H*_E_	UH_e_	*F*_IS_ ^a^	Private alleles (Population)	
								Locus (*N* ^b^)	PAr
AVE	21.000	1.953	2.630	0.324	0.356	0.364	0.113*		0.000
MOF	18.000	1.956	2.630	0.306	0.359	0.369	0.176**	A37 (256)^d^	0.060
BAI	19.800	1.946	2.790	0.383	0.402	0.413	0.059		0.030
LAU	23.900	1.946	2.590	0.369	0.376	0.384	0.034		0.000
SME	14.900	1.994	2.960	0.399	0.374	0.387	−0.042		0.110
CAV	19.800	1.993	2.720	0.406	0.384	0.394	−0.050		0.030
CHI	20.000	2.036	2.820	0.440	0.379	0.388	−0.137	AG10 (213)^d^	0.060
RAC	17.800	2.097	2.890	0.388	0.413	0.425	0.069		0.030
MAG	19.800	2.015	2.530	0.391	0.369	0.379	−0.049	AG13 (248)^d^	0.050
SAS	15.900	2.118	3.060	0.393	0.379	0.392	−0.013	AG10 (238), alng4 (128)^d^	0.160
ANZ	14.000	2.082	3.010	0.450	0.409	0.424	−0.062	AG10 (215)^d^, AG10 (240)	0.210
SEV	10.000	1.860	2.900	0.420	0.381	0.401	−0.050		0.000
NUP	17.800	2.039	2.850	0.340	0.374	0.385	0.103		0.060
BUO	17.000	1.879	2.760	0.359	0.363	0.374	0.041	alng4 (146)	0.080
VIT	14.500	1.759	2.610	0.310	0.327	0.338	0.072		0.000
ORS	18.900	1.832	2.570	0.311	0.330	0.339	0.084		0.000
SCA	18.900	1.746	2.680	0.326	0.316	0.325	−0.004		0.020
CET	19.900	1.927	2.720	0.361	0.383	0.393	0.074		0.010
FIU	25.000	1.944	2.760	0.308	0.325	0.331	0.071		0.010
MAC	23.600	1.899	2.850	0.366	0.380	0.388	0.036		0.030
FOR	18.700	1.816	2.470	0.384	0.361	0.371	−0.044		0.010
STA^c^	5.000	1.295	(1.700)	0.200	0.192	0.213	0.069		(0.000)
SBR	20.000	1.863	2.670	0.345	0.342	0.351	0.017		0.010
Mean	18.009 (4.390)	1.913 (0.168)	2.749 (0.158)	0.360 (0.054)	0.360 (0.045)	0.371 (0.044)			0.044 (0.055)

^a^ Significance of inbreeding coefficient *F*_IS_ was tested using a nonparametric approach described by Excoffier et al. (2005) with 1000 permutations: *p < 0.05, **p < 0.01, ***p < 0.01.

^b^ Number of private alleles within the respective locus.

^c^ Allelic richness and private allelic richness were not standardized but computed on the minimum sample size of five individuals.

^d^ SSR allele shared with *A. glutinosa* species.

The overall *F*_IS_ did not differ significantly from zero except for two populations, AVE and MOF. In these alder stands, *F*_IS_ values were large and positive, suggesting that inbreeding in Italian alder trees might have occurred in the northernmost regions of *A. cordata* distribution (Table 2). Conversely, *F*_IS_ was negative for 9 out of the 23 alder populations, indicating a slight surplus of heterozygotes in three sampling sites from NP Cilento—Vallo di Diano (SME, CAV, CHI), three from NP Appennino Lucano Val d'Agri Lagonegrese (MAG, SAS, ANZ), and three from NP Pollino (SEV, SCA) and RP of Serre (FOR) (Table 2). In addition, Wilcoxon's signed‐rank test revealed a significant (*p* < 0.05) recent reduction in effective population size only for FOR (heterozygote excess) and STA (heterozygote deficiency), which also showed deviation from the typical L‐shaped distribution of allele frequencies (Table S6). Using the *M* ratio test, five of the alder populations, MOF, BAI, LAU, SME, and STA, displayed a genetic signature consistent with a bottleneck. Their *G*–*W* values ranged from 0.59 (SME) to 0.67 (MOF), which is lower than the critical threshold (0.68) (Table S6).

### 3.3 Spatial Genetic Structure of Italian Alder Populations

**FIGURE 4:**
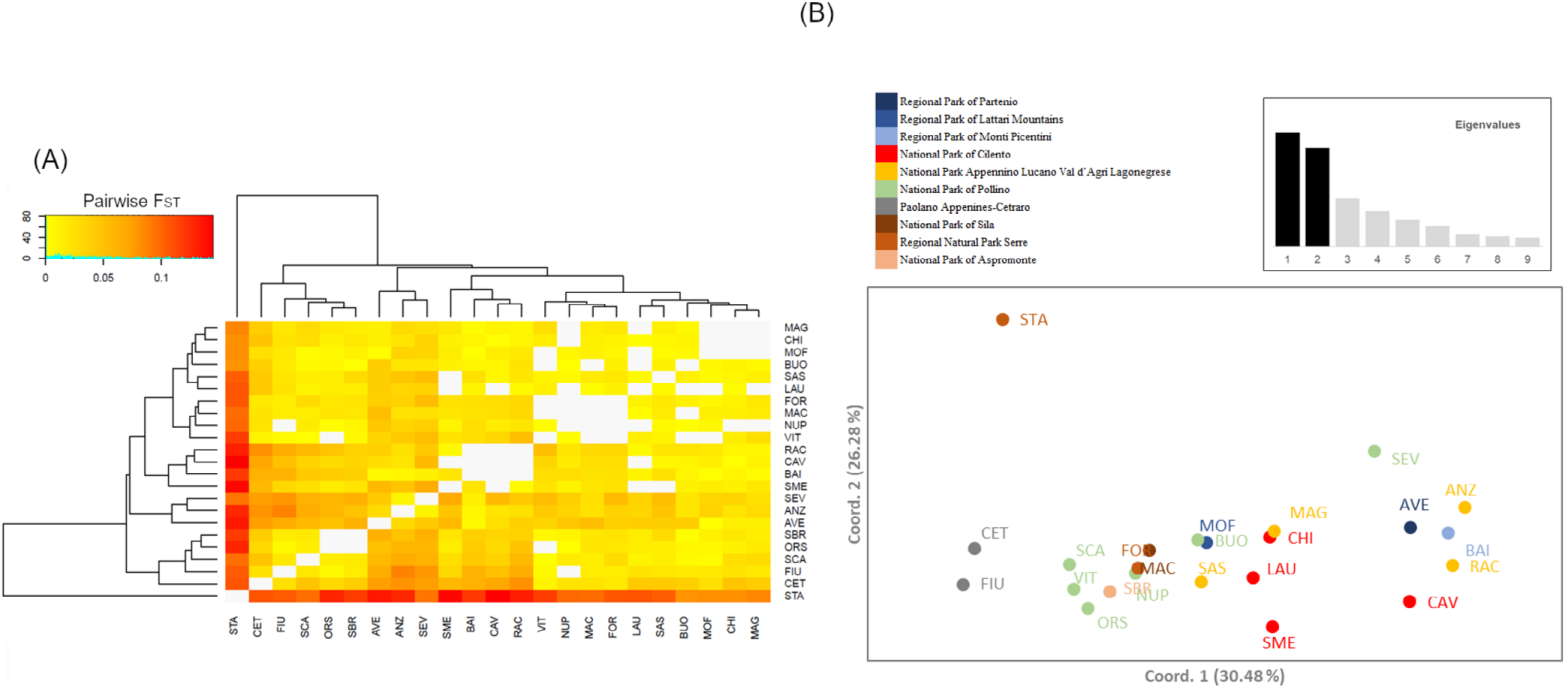
Heatmap (A) and Principal Coordinate Analysis (B) of pairwise *F*_ST_ values between 23 Italian alder populations collected in the Paolano Appenines and nine protected macro areas of Southern Italy based on SSR markers. Dendrograms were plotted using the unweighted‐pair group method with arithmetic mean (UPGMA).

Overall, genetic differentiation was highly statistically significant but extremely low among *A. cordata* populations (*F*_SC_ = 1.55%, *p* < 0.001) and among protected/unprotected macro‐areas (*F*_CT_ = 0.85%, *p* = 0.0176). AMOVA analysis showed that most of the variation was found among individuals within populations (*F*_IS_ = 2.49%, *p* = 0.0078) and within individuals (*F*_IT_ = 95.07%, *p* < 0.001) (Table S7). Pairwise *F*_ST_ among populations was significant in 11.07% of the comparisons after Bonferroni correction, with a mean value of 0.03 ± 0.031. The highest *F*_ST_ values were detected between STA (*F*_ST_ = 0.079–0.144) and the remaining Italian alder collections. Although the PCoA of all 417 individuals in the 23 populations accounted for 30.48% (axis 1) and 26.28% (axis 2) of the total variance based on pairwise *F*_ST_ index, neither the heatmap nor PCoA displayed a sharp genetic partitioning of the *A. cordata* trees across the nine protected geographic macro‐areas across Southern Italy (Figure 4). Only STA of the RP of Serre is genetically distant from the remaining 22 Italian alder populations. Indeed, STRUCTURE indicated *K* = 1 as the most appropriate number of population groups because the highest Log‐likelihood value of data *L*(*K*) as a function of *K* averaged over six replicates was detected at *K* = 1 (Figure S3). While still confirming the lack of clear population genetic structure, the UPGMA tree based on Nei's genetic distance also provided additional insights into the relationships among *A. cordata* trees (Figure S4). Despite the low‐estimated *F*_ST_, 10 of 11 populations sampled across Calabria Region showed a tendency to group together (Figure S4). The Mantel correlation between the pairwise linearized genetic differentiation values [*F*_ST_/(1 − *F*_ST_)] and the spherical geographic distances between the sampling sites was significant but low (*r* = 0.330, *p* = 0.025), indicating an overall absence of isolation by distance among *A. cordata* populations (Figure S5A). The Mantel Bearing correlogram analysis showed in fact a significant positive spatial autocorrelation between *A. cordata* trees only in the first ring of the northeast direction, corresponding to a maximum distance of 33.021 km (Figure S5B).

### 3.4 In Situ and Ex Situ Conservation Priority of Italian Alder Populations

**FIGURE 5:**
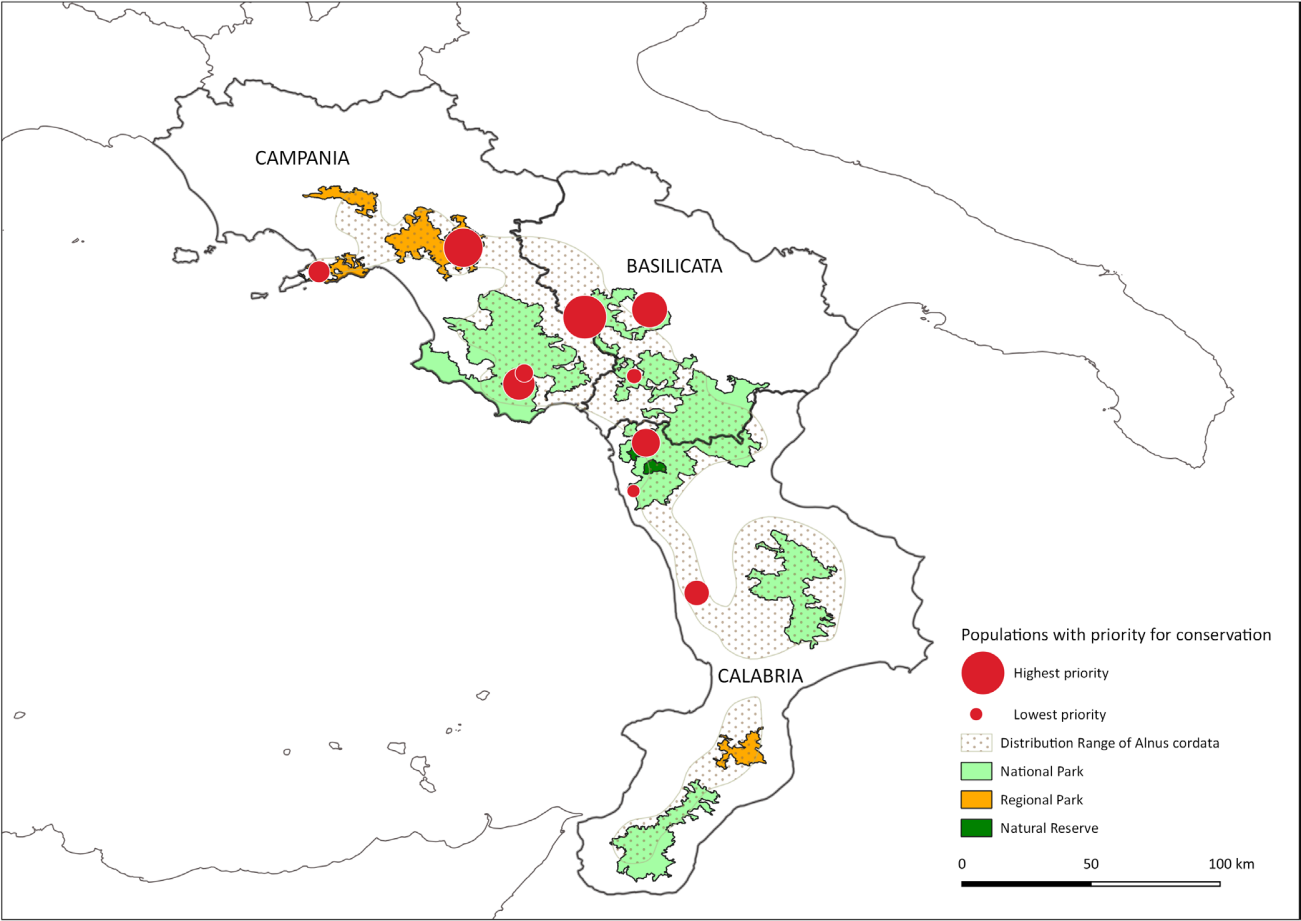
Geographic areas with the highest in situ conservation priority for *A. cordata* species across 10 macro areas including five National Parks and three Regional Parks of Southern Italy.

**FIGURE 6:**
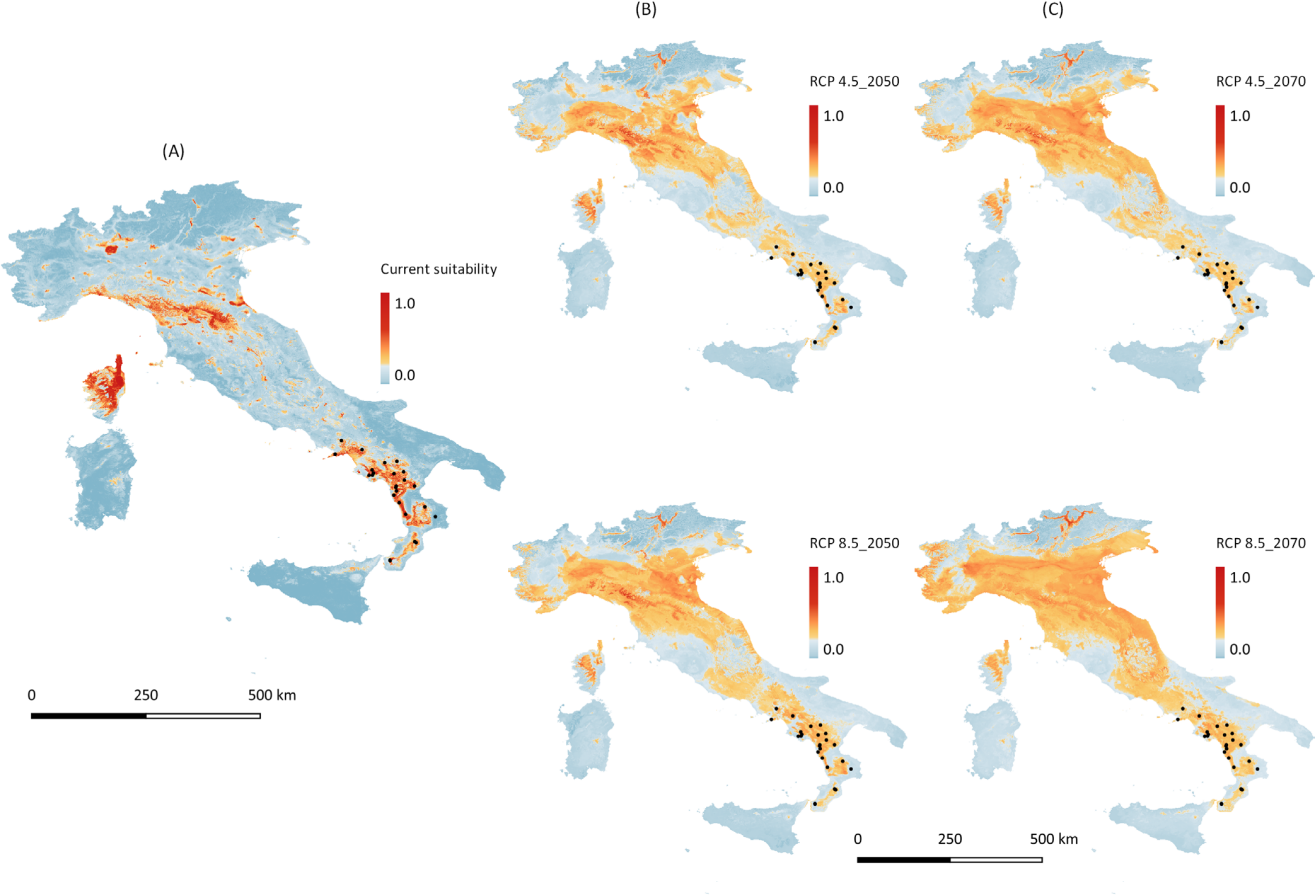
(A) Predicted current and (B) future potential geographic distribution of *Alnus cordata* by 2050 (range 2041–2060) and (C) 2070 (range 2061–2080), according to the climate CMIP6 model under RCP 4.5 and RCP 8.5 scenarios across nine protected areas and Paolano Appenines. Italian alder populations used for the SSR genotyping analysis were also reported (black dot).

The application of reserve selection procedure implemented in DIVA‐GIS software demonstrated that 10 populations are needed (~43%) to cover 100% of the genetic diversity of Italian alder (Figure 5). A total of five populations, BAI, CHI, SAS, ANZ, and NUP, were categorized as a high priority for in situ conservation. Conversely, five *A. cordata* sites, MOF, SME, MAG, BUO, and FIU, were classified as relevant for *A. cordata* conservation but with low priority. All alder stands included in the priority list are located inside regional or national protected areas of the south‐central Apennines, except FIU (Figure 5). The SDM developed for the potential distribution of the *A. cordata* across the Italian Peninsula and the nearby Corsica Island was characterized by high mean predictive accuracy, showing AUC = 0.991 and TSS = 0.906. Spatial congruence between the predicted model and the current known distribution of *A. cordata* across the native range of Southern Italy and Corsica Island (Euforgen database) was detected (Figure 6). In contrast, our results suggested that habitat suitability is also high in the Tusco‐Emilian Apennines. The most important environmental variables (variance ≥ 10%) in the SDM were the mean temperature of driest quarter (BIO9), precipitation of coldest quarter (BIO19), temperature seasonality (BIO4), precipitation of warmest quarter (BIO18), and isothermality (BIO3) (Figure S6). The current potential distribution analysis revealed that from moderate to high potential habitat suitability was dominant for all 10 alder populations with priority conservation (Table 3, Figure 6). The comparison between the present distribution model and future projections for climatic layers CIMP6_2050 and CIMP6_2070 using RCP 4.5 and RCP 8.5 indicated a potential climate‐driven range shift of habitat suitability for *A. cordata* from Southern Italy to Po Valley in the Northern regions (Figure 6). Among the 10 selected sites, only FIU is expected to demonstrate moderate potential suitability for *A. cordata* distribution. Under the CIMP6 2070 RCP 4.5 and RCP 8.5 scenarios, both models predicted that 100% of alder trees sampled at key site ANZ had no climatic suitability. None of the total 417 *A. cordata* trees were forecasted to have moderate or high habitat suitability across the sampling sites (Table 3, Figure 6).

**TABLE 3:** The percentage of Italian alder trees located in the projected areas of *A. cordata* under current and future (2050 and 2070) climate change scenarios of CIMP6 (RCP 4.5 and RCP 8.5) categorizing in Not, Low, Medium, High suitability value.

Pop	Current suitability				RCP 4.5_2050 suitability				RCP 4.5_2070 suitability				RCP 8.5_2050 suitability				RCP 8.5_2070 suitability			
	Not	Low	Mod	High	Not	Low	Mod	High	Not	Low	Mod	High	Not	Low	Mod	High	Not	Low	Mod	High
AVE	0.00	0.00	9.52	90.48	33.33	66.67	0.00	0.00	33.33	66.67	0.00	0.00	0.00	100.00	0.00	0.00	9.52	90.48	0.00	0.00
MOF	0.00	0.00	0.00	100.00	0.00	100.00	0.00	0.00	0.00	100.00	0.00	0.00	0.00	100.00	0.00	0.00	0.00	100.00	0.00	0.00
BAI	0.00	0.00	100.00	0.00	0.00	100.00	0.00	0.00	0.00	100.00	0.00	0.00	0.00	100.00	0.00	0.00	0.00	100.00	0.00	0.00
LAU	0.00	0.00	0.00	100.00	0.00	100.00	0.00	0.00	0.00	100.00	0.00	0.00	0.00	100.00	0.00	0.00	0.00	100.00	0.00	0.00
SME	0.00	0.00	20.00	80.00	0.00	100.00	0.00	0.00	0.00	100.00	0.00	0.00	100.00	0.00	0.00	0.00	0.00	100.00	0.00	0.00
CAV	0.00	0.00	0.00	100.00	0.00	100.00	0.00	0.00	0.00	100.00	0.00	0.00	100.00	0.00	0.00	0.00	0.00	100.00	0.00	0.00
CHI	0.00	0.00	52.63	52.63	0.00	100.00	0.00	0.00	0.00	100.00	0.00	0.00	0.00	100.00	0.00	0.00	0.00	100.00	0.00	0.00
RAC	0.00	72.22	27.78	0.00	100.00	0.00	0.00	0.00	100.00	0.00	0.00	0.00	0.00	100.00	0.00	0.00	100.00	0.00	0.00	0.00
MAG	0.00	0.00	0.00	100.00	0.00	100.00	0.00	0.00	0.00	100.00	0.00	0.00	0.00	100.00	0.00	0.00	0.00	100.00	0.00	0.00
SAS	0.00	0.00	0.00	100.00	0.00	100.00	0.00	0.00	0.00	100.00	0.00	0.00	0.00	100.00	0.00	0.00	0.00	100.00	0.00	0.00
ANZ	0.00	0.00	100.00	0.00	100.00	0.00	0.00	0.00	100.00	0.00	0.00	0.00	100.00	0.00	0.00	0.00	100.00	0.00	0.00	0.00
SEV	0.00	0.00	0.00	100.00	0.00	100.00	0.00	0.00	0.00	100.00	0.00	0.00	0.00	100.00	0.00	0.00	0.00	100.00	0.00	0.00
NUP	0.00	0.00	33.33	66.67	50.00	50.00	0.00	0.00	88.89	11.11	0.00	0.00	83.33	16.67	0.00	0.00	38.89	44.44	0.00	0.00
BUO	0.00	0.00	94.12	5.88	0.00	100.00	0.00	0.00	0.00	100.00	0.00	0.00	5.88	94.12	0.00	0.00	5.88	94.12	0.00	0.00
VIT	0.00	0.00	0.00	100.00	100.00	0.00	0.00	0.00	100.00	0.00	0.00	0.00	100.00	0.00	0.00	0.00	100.00	0.00	0.00	0.00
ORS	0.00	0.00	100.00	0.00	36.84	63.16	0.00	0.00	100.00	0.00	0.00	0.00	100.00	0.00	0.00	0.00	42.11	57.89	0.00	0.00
SCA	0.00	0.00	0.00	100.00	0.00	100.00	0.00	0.00	0.00	100.00	0.00	0.00	0.00	100.00	0.00	0.00	105.26	0.00	0.00	0.00
CET	0.00	0.00	15.00	85.00	0.00	100.00	0.00	0.00	0.00	100.00	0.00	0.00	0.00	100.00	0.00	0.00	0.00	100.00	0.00	0.00
FIU	0.00	0.00	0.00	100.00	0.00	84.00	16.00	0.00	0.00	100.00	0.00	0.00	0.00	100.00	0.00	0.00	0.00	100.00	0.00	0.00
MAC	0.00	0.00	37.50	62.50	0.00	100.00	0.00	0.00	16.67	83.33	0.00	0.00	0.00	100.00	0.00	0.00	37.50	79.17	0.00	0.00
FOR	0.00	0.00	0.00	100.00	0.00	100.00	0.00	0.00	0.00	100.00	0.00	0.00	0.00	100.00	0.00	0.00	100.00	0.00	0.00	0.00
STA	0.00	0.00	100.00	0.00	0.00	100.00	0.00	0.00	0.00	100.00	0.00	0.00	0.00	100.00	0.00	0.00	100.00	0.00	0.00	0.00
SBR	0.00	0.00	0.00	100.00	100.00	0.00	0.00	0.00	100.00	0.00	0.00	0.00	100.00	0.00	0.00	0.00	100.00	0.00	0.00	0.00

*Note:* Alder stands included in the priority list inferred by the reserve selection procedure implemented in DIVA‐GIS software were shaded in gray.

## 4 Discussion

### 4.1 Genetic Diversity of *A. cordata* Species

In agreement with narrow endemic tree species such as *Pinus nigra* subsp. *laricio* (Bonavita et al. 2016; Scotti‐Saintagne et al. 2024) and *P. heldreichii* (Piovesan et al. 2023), whose geographic distribution is restricted to the Southern Italian Apennines and Corsica Island, *A. cordata* showed moderate genetic diversity levels across its Italian native range. The mean unbiased heterozygosity of *A. cordata* was in fact lower compared to that reported for widely distributed European alder species such as *A. glutinosa* (Havrdová et al. 2015) but similar to *A. incana* (Mandák et al. 2016) investigated using the same SSR array. Indeed, the statistically significant positive relationship between the latitude of the sampled sites and *R*_s_ suggests that the genetic diversity of *A. cordata* tends to decline towards the latitudinal range margins of the Italian peninsula, aligning with the central‐marginal hypothesis proposed by Eckert et al. (2008). The Italian alders situated in the Massifs of Pollino & Orsomarso (VIT, ORS, and SCA) and Aspromonte area (SBR) at its southern margins and the Lattari (MOF) and Partenio (AVE) Mountains at its northernmost edge display the lowest levels of genetic diversity. In addition, significantly positive values of inbreeding (*F*_IS_) were observed in the Italian alder populations sampled in the northernmost regions of *A. cordata* distribution (MOF and AVE). Clear signals of recent reduction in effective population size were also observed in the peripheral RP of Serre. We detected signatures of ancient bottlenecks in five Italian alder populations MOF, BAI, LAU, SME, and STA. The presence of alder wood exploitation has been documented throughout Southern Italy (Ducci and Tani 2009). However, formulating unequivocal conclusions regarding the role of humans in shaping the current pattern of alder diversity remains challenging. We hypothesize that Italian alders at the edges of their range may have experienced increased inbreeding and stochastic reduction of genetic diversity over extended periods. In particular, Italy faces a significant number of wildfire events every year, with a marked latitudinal gradient in fire regimes from North to South (Elia et al. 2020)—particularly in the regions of Calabria and Sicily, followed by Campania and Sardinia (Malandra et al. 2022), even within protected areas (Cillis et al. 2023). These disturbances may further contribute to the fragmentation of natural stands and human‐driven population bottlenecks, especially when compared to more centrally located populations.

Approximately 50% of Italian alder populations located between 40°50′0.3″ N and 40°2′56.040″ N exhibited notably high levels of UH_e_ and *R*_s_. These peaks of genetic diversity coincide with the macro‐region of the Italian Peninsula where *Alnus* spp. survived after the Last Glacial Maximum (LGM), as evidenced by fossil pollen data detected in Monticchio lakes (Douda et al. 2014). Alnus sp. presence during the LGM (26–14 cal kyr BP) and into the Early Holocene was recorded in two Monticchio lakes, Lago Grande and Lago Piccolo, situated at 656 and 657 m above sea level, respectively, within a crater on the western side of the Monte Vulture complex in the Basilicata region. As many forest tree species in the Northern Hemisphere, glacial refugia served as reservoirs of allelic richness compared to areas colonized after the last ice age (Krebs et al. 2004). Our study reveals that regions with high allelic richness, such as the NP of Appennino Lucano Val d'Agri Lagonegrese, are in proximity to ancient pollen deposits. These findings may support the persistence of *Alnus cordata* in scattered plots within the Southern Italian Apennines during the cold and dry climate of the Pleistocene. However, our data suggest that the evolutionary scenario is more complicated, and additional process could influence the observed patterns of *A. cordata* genetic diversity in the central core of distribution. The recognition of cytoplasmic captures and introgressive hybridization events over time (King and Ferris 2000; Gryta et al. 2017) suggests the presence of permeable boundaries between sympatric alder species across Europe. Consistent with Villani et al. (2021), our findings confirmed that the co‐occurrence of *A. cordata* with *A. glutinosa* below 1000 m above sea level, particularly along riverbanks, lake shores, and marshy waterlogged sites, facilitated long‐term interspecific hybridization and introgression of neutral SSR alleles between these species in sympatric sites across the Southern Apennines. Similarly, we documented hybridization/backcross between *A. cordata* and *A. glutinosa* in three mixed populations—SME, SEV, and VIT—located in the NP Cilento–Vallo di Diano and NP Pollino. After excluding hybrids and backcrosses from our dataset, we still detected a statistically significant positive correlation between the admixture coefficient with *A. glutinosa* and *R*_s_/PAr of *A. cordata* populations. In addition to the findings reported by Villani et al. (2021), our results indicate that ancient and ongoing introgressive hybridization with *A. glutinosa* increases the genetic diversity in *A. cordata* populations where the alder species coexist. This locally increments of the private allelic richness do not affect the spatial genetic structure of *A. cordata* populations across the Italian native range (see below) but is able to in part mitigate the risk of pronounced genetic erosion typical of endemic trees with restricted distribution range as *A. cordata* species (Scotti‐Saintagne et al. 2024). As already reported for representatives of the genus *Pinus* (Szczepański et al. 2024) and *Populus* (Meirmans et al. 2017), contemporary hybridization appears to have a geographically localized effect on the genetic variation of *A. cordata* populations. Hybridization is generally considered an important source of genotypic novelty in tree evolution (Burke and Arnold 2001), and the introgression of adaptive genetic variation has been extensively documented in several forest tree species (Suarez‐Gonzalez et al. 2018). However, our data, based on neutral nuclear SSR markers, are not suitable for detecting adaptive variation or evaluating whether hybridization facilitates the adaptation of *A. cordata* to diverse habitats. Ongoing genomic and ecological studies aim to investigate the potential effects of introgression with *A. glutinosa* and its possible role in local adaptation of *A. cordata* populations.

### 4.2 IBD and Landscape Effect on *A. cordata* Population Structure

In this study, the Mantel Bearing correlogram analysis did not support an isolation‐by‐distance model of gene flow for *A. cordata* , except for geographically close populations (within ~33 km) in the northeastern part of its Italian native range. We observed relatively low levels of genetic differentiation among *A. cordata* populations, indicating moderate to high gene flow across the Italian peninsula. These findings are consistent with those reported for several highly outcrossing and wind‐pollinated alder species, such as *A. glutinosa* (Havrdová et al. 2015; Mingeot et al. 2016) and *A. incana* (Mandák et al. 2016). In contrast to the central‐marginal hypothesis proposed by Eckert et al. (2008), we did not detect significant genetic differentiation between geographically central and peripheral *A. cordata* populations across Southern Italy, except for the STA site located within the NP Serre. Bayesian inference analysis suggests that the significant genetic divergence observed between the STA population and the broader alder gene pool may not solely reflect biological patterns, but could also be influenced by the limited sample size of the STA population. As observed in many long‐lived tree species (Miguel‐Peñaloza et al. 2023), it is reasonable to presume that mechanisms fostering the reduction of genetic diversity within peripheral populations of *A. cordata* , such as habitat fragmentation and recent bottlenecks, may not be significantly strong enough to shape the spatial genetic structure between central and peripheral alder populations. Ducci and Tani (2009) suggested that the absence of natural barriers in Southern Italy promotes extensive gene flow among *Alnus cordata* populations through both pollen and seeds, primarily dispersed by wind and water. This genetic connectivity may have been further enhanced by human‐mediated long‐distance dispersal of Italian alder germplasm across Italy, often selected for its ecological value and pioneering ability (Danise et al. 2021). However, we can also add that the actinorhizal symbiosis with *Frankia* sp., a nitrogen‐fixing filamentous bacterium, plays a critical role in the ability of alder species to colonize ecologically marginal, degraded, or nutrient‐deficient sites (Mallet and Roy 2014; Buchbauerová et al. 2024). This symbiosis enables *A. cordata* to establish and persist in a wide range of ecologically diverse or challenging environments, potentially enhancing its resilience to habitat fragmentation.

### 4.3 In Situ and Ex Situ Conservation of *A. cordata* Facing the Climate Change

An effective conservation strategy, as formulated by Maxted et al. (2008), aims to maximize the overall gene pool, whether conserved in situ or ex situ, in a complementary manner, and make it available for potential or actual utilization. Our genetic diversity assessment suggests that a significant portion of the Italian alder's genetic diversity is effectively conserved using in situ techniques. Out of 10 *A. cordata* populations with significant priority for conservation, nine are currently included in existing protected areas, regional (Lattari and Picentini Mountains) or NPs (Cilento—Vallo di Diano, Appennino Lucano Val d'Agri Lagonegrese and Pollino) of the south‐central Apennines. We have identified a “conservation gap element” subjected to human pressures, the unprotected FIU stand of *A. cordata* , which is located on Cucuzzo mountain near the Tyrrhenian Sea, the highest peak in the coastal chain of the Paolano Apennines. It is noteworthy that a basic requirement for proper genetic management of forest trees calls for the establishment of a sufficiently large number of individual trees to maintain adaptive potential across generations (Koskela et al. 2013). This reduces the risk of inbreeding depression and drift during tree evolution. Our study suggests the need for special efforts to further protect four natural stands of *A. cordata* , classified as relevant for conservation. These stands are experiencing size reduction and signs of inbreeding events. They are situated in the RP Lattari Mountains, RP Picentini Mountains, NP Cilento–Vallo di Diano, and NP Appennino Lucano Val d'Agri Lagonegrese. Finally, the SDM analysis and model projections under future scenarios revealed a severe reduction in habitat suitability of *A. cordata* in approximately 50 years, especially in the native range of Southern Italy. In accordance with Borghetti et al. (1989), mild winters and high rainfall levels during the driest and coldest part of the year are the most important factors that determine the current habitat suitability of *A. cordata* . According to the current prediction results, most of the suitable distribution regions of *A. cordata* were mainly in the native range of Southern Italy and Corsica but also in the Tusco‐Emilian Apennines of Northern Italy. As already forecasted for several plants (Hansson et al. 2021), the increase in temperature and the decrease of the rainfall levels, mostly during the driest quarter, should force *A. cordata* ecosystems to shift to higher elevations in restricted areas of the Southern margin and also expand its distribution eastward towards the summer‐rainfall regions of Po Valley in Northern Italy by 2070 in both RCP 4.5 and RCP 8.5 scenarios. In response to contemporary climate change, alder trees can adapt in situ, migrate, or die (Christmas et al. 2016). Successful migration via natural dispersal or adaptation in situ of *A. cordata* can now only be postulated. The intrinsic capacity of *A. cordata* to adapt to future climatic scenarios is currently under evaluation in our lab using functional genomics data combined with the detection of adaptively relevant phenotypic variation within alder populations. However, since *A. cordata* is projected to experience a decline in habitat suitability in its native range of Southern Italy, assisted migration across the partial geographic gaps of Central Italy might be necessary for proper conservation (Stanturf et al. 2024). Nonetheless, this study also highlighted that, under this scenario and given the wide distribution range of *A. glutinosa* in Italy, there is a plausible risk of increased hybridization between *A. cordata* and *A. glutinosa* in the near future, with potential consequences for conservation genetics. Careful consideration is therefore essential to ensure assisted migration does not inadvertently exacerbate the risk of hybridization. Therefore, we propose the establishment of an ex situ conservation program for Italian alder trees (i.e., seed bank or germplasm core collection) in all prioritized sites, in particular for ANZ and NUP sites, showing no climatic suitability for 100% and ≥ 50% of alder trees in the near future (scenarios RCP 4.52050 and RCP 8.52050).

In conclusion, our study highlights the importance of integrating genetic analyses, habitat suitability modeling, and spatial prioritization techniques for effective conservation planning in the face of climate change. While we recognize that some populations of *A. cordata* are underrepresented in our dataset due to sampling challenges, the identification of areas with high genetic diversity and future habitat suitability offers preliminary guidance for developing targeted conservation strategies and ensuring the long‐term viability of *A. cordata* populations within their native Italian range.

## Author Contributions

**Paola Pollegioni:** conceptualization (lead), data curation (equal), formal analysis (equal), funding acquisition (lead), investigation (lead), methodology (lead), project administration (lead), software (equal), supervision (equal), validation (equal), visualization (supporting), writing – original draft (lead), writing – review and editing (lead). **Alexis Marchesini:** conceptualization (equal), data curation (equal), formal analysis (equal), investigation (equal), methodology (equal), software (equal), writing – original draft (equal), writing – review and editing (equal). **Muriel Gaudet:** data curation (supporting), investigation (supporting), methodology (supporting), writing – review and editing (supporting). **Francesca Chiocchini:** data curation (supporting), methodology (supporting), software (equal), visualization (lead), writing – review and editing (equal). **Flavio Monti:** data curation (supporting), methodology (supporting), software (equal), visualization (supporting), writing – review and editing (equal). **Luca Leonardi:** investigation (supporting), methodology (supporting), writing – review and editing (supporting). **Marcello Cherubini:** formal analysis (supporting), investigation (supporting), methodology (supporting), writing – review and editing (supporting). **Claudia Mattioni:** conceptualization (equal), data curation (equal), formal analysis (equal), funding acquisition (equal), investigation (equal), methodology (equal), project administration (equal), resources (equal), supervision (equal), validation (equal), writing – original draft (equal), writing – review and editing (equal).

## Conflicts of Interest

The authors declare no conflicts of interest.

## Supporting information


**Data S1:** ece372018‐sup‐0001‐DataS1.pdf.

## Data Availability

Raw data for SSR markers and occurrence points of *A. cordata* for Species Distribution Modeling (SDM) analysis are available at Dryad database DOI: 10.5061/dryad.931zcrjtk.
