# Supplementary material for: What Shapes the Genetic Diversity of the Alnus cordata Species Across Its Italian Native Range? Informing Conservation Strategies
Source: Ecol Evol. 2025 Aug 31;15(9):e72018. doi: 10.1002/ece3.72018 (PMC12399267; doi:10.1002/ece3.72018)
Supplement: Supplementary file 1 — Data S1: ece372018‐sup‐0001‐DataS1.pdf. [file ECE3-15-e72018-s001.pdf]

Supplemental Information_1 for:

What shapes the genetic diversity of the Alnus cordata species across its Italian native range? informing conservation strategies

Paola Pollegioni, Alexis Marchesini, Muriel Gaudet, Francesca Chiocchini, Flavio Monti, Luca Leonardi, Marcello Cherubini, Claudia Mattioni,

Table of Contents:

**Appendix S1**	Page 2
**Table S1**	Page 4
**Table S2**	Page 6
**Table S3**	Page 8
**Table S4**	Page 9
**Table S5**	Page 9
**Table S6**	Page 10
**Table S7**	Page 11
**Fig. S1**	Page 12
**Fig. S2**	Page 13
**Fig. S3**	Page 14
**Fig. S4**	Page 15
**Fig. S5**	Page 16
**Fig. S6**	Page 17
**References**	Page 18

Appendix S1

1. **Genetic diversity of the SSR markers**

Data Analysis

Descriptive gene diversity parameters, number of observed alleles (A), effective number of alleles (Ne), observed (Ho), and expected (H_E) heterozygosity, polymorphic information content (PIC), and the estimated null allele frequency were calculated at each locus and over all loci of *A. cordata* and *A. glutinosa* germplasm as described by Pollegioni et al (2014). Similarly, allele dropout and null alleles were tested for each locus using FreeNa software (Chapuis & Estoup, 2007). In addition, the exact tests for genotypic linkage disequilibrium for each pair of SSR loci were also performed with GENEPOP version 4.7 (Rousset 2008) using 10,000 iterations and Bonferroni correction.

Results for *A. cordata* species

Out of ten SSR loci used in the present study, nine (A2, A22, A35, A37, A38, AG10, AG13, alma11, and alng4) were polymorphic in the sampled populations of *A. cordata*. A total of 57 alleles were detected in the 417 Italian alder trees genotyped. The number of alleles per locus ranged from three at locus A35, and A38 to a maximum of 11 at locus AG10 and alng4, with an average of 5.700 ± 3.622. The observed heterozygosity and gene diversity greatly varied across the nine SSR loci. The average observed (HO) and expected (HE) heterozygosity were 0.400 (SE=0.241) and 0.400 (SE=0.222), respectively. Highly significant ($p<0.001$) departures from Hardy–Weinberg expectations across all samples were found for A37 and alng4 using a likelihood ratio (G test) procedure, corresponding to f(F_{IS}) significantly greater than zero. These data indicated a high level of heterozygote deficiency probably because of the allelic dropout including presence of null alleles. Most of the loci showed in fact no signs of null allele presence ($F_{nul} < 0.1$) except A37, and alng4 characterized by the highest prevalence of null alleles with F_{nul} ranging from 0.1368 for alng4 to 0.2582 for A37 (Table S2). Furthermore, analysis using FreeNa indicated that none of these SSR loci displayed conclusive evidence of null alleles (estimated null allele frequency for each SSR ranged from 0.15 to 0.3 in less than 30% of populations). Indeed, population genetic differentiation F_{ST} was significant ($P < 0.05$) for three SSR loci, A2, A37, and A22. After ENA correction, values of F_{ST}(null) for each locus were only slightly different from F_{ST} values. Finally, no statistically significant evidence of linkage disequilibrium has been found between nine polymorphic SSR loci.

Results for *A. glutinosa* species

All ten SSR loci used in the present study were polymorphic in the sampled populations of *A. glutinosa* (for more details for *A. glutinosa* trees please see Table S2). A total of 91 alleles were

detected in the 96 *A. glutinosa* trees genotyped. The number of alleles per locus ranged from three at locus A2, to a maximum of 16 at locus alma11, with an average of 9.100 ± 4.458. The observed heterozygosity and gene diversity greatly varied across the ten SSR loci. The average observed (HO) and expected (HE) heterozygosity were 0.547 (SE=0.228) and 0.642 (SE=0.224), respectively. Highly significant ($p<0.001$) departures from Hardy–Weinberg expectations across all samples were found for A37, and alng4 using a likelihood ratio (G test) procedure, corresponding to f(F_{IS}) significantly greater than zero. These data indicated a high level of heterozygote deficiency probably because of the allelic dropout including presence of null alleles. Similar to *A. cordata* results, most of the loci showed in fact no signs of null allele presence ($F_{nul} < 0.1$) except A37, and alng4 characterized by the highest prevalence of null alleles with F_{nul} ranging from 0.1218 for A37 to 0.3801 for alng4 (Table S2). Furthermore, analysis using FreeNa indicated that none of these SSR loci displayed conclusive evidence of null alleles (estimated null allele frequency for each SSR ranged from 0.15 to 0.3 in less than 30% of populations). Indeed, population genetic differentiation F_{ST} was highly significant ($P < 0.001$) for four SSR loci, A38, AG10, alma1 and alma11. After ENA correction, values of F_{ST}(null) for each locus were only slightly different from F_{ST} values. Finally, no statistically significant evidence of linkage disequilibrium has been found between ten SSR loci.

Supplementary Tables

Table S1: Summary of the BLASTn similarity searches conducted on ten nuclear microsatellite sequences of *Alnus glutinosa* (Mingeot et al. 2010; Lepais et al. 2011; Drašnarová et al. 2014) against the *Alnus glutinosa* genome assembly (dhAlnGlut1) provided by the Darwin Tree of Life Project (https://www.darwintreeoflife.org/). Among the ten microsatellites analyzed, seven were successfully cross amplified to *A. glutinosa* from four closely related species: *Betula maximowicziana* (Tsuda et al. 2009a, b), *Betula platyphylla* (Wu et al. 2002), *Corylus avellana* (Gürcan et al. 2010), and *Alnus maritima* (Lance et al. 2009).

Locus name	Original Species (GenBank) [a]	Genbank [b]	BLAST Result for SSR fragment	Repeat Motif in *A.glutinosa*	Alignment Length (bp)	E-value	Identity (%)	Gene Function	Source
A35	BETMAX (CD276907)	KF724876	*Alnus glutinosa* Chr9 1,571,628-1,574,550	(TC)16*	139	2e-34	88	Uncharacterized gene LOC133877396	Tsuda et al. 2009a
A37	BETMAX (CD277113)	KF724878	*Alnus glutinosa* Chr2 27,426,234-27,428,853	(TC)10*	422	5e-171	93	Small ribosomal subunit protein uS7-like LOC133859134	Tsuda et al. 2009b
A38	BETMAX (CD278280)	KF724880	*Alnus glutinosa* Chr3 34,892,384-34,893,756	(CAA)8*	369	2e-137	92	Uncharacterized gene LOC133862301	Tsuda et al. 2009a
A2	BETPLA (AB084474)	KF724864	*Alnus glutinosa* Chr1 18,122,111-18,122,253	(GT)8GAAAGC(GA)2A	155	2e-43	85	acetolactate synthase small subunit 1, chloroplastic-like gene LOC133875775	Wu et al. 2002
A22	CORAVE (FJ986523)	KF724872	*Alnus glutinosa* Chr2 38,769,107–38,769,202	(AG)13	96	6e-40	99	(AG)13 in the promotor region of zinc finger protein JAGGED gene LOC133860621	Gürcan et al. 2010
AG10	AGLUT (JF313811)	JF313811	*Alnus glutinosa* Chr6 19,993,18–19,993,396	(TC)11T(G)4(T)4	213	2e-104	99	nucleotide-sugar uncharacterized transporter 1 LOC133870038	Lepais et al. 2011
AG13	AGLUT (JF313814)	JF313814	*Alnus glutinosa* Chr13 4,300,258–4,300,515	(TG)12	262	2e-121	98	uncharacterized gene [enables nucleic acid binding] LOC133854778	Lepais et al. 2011
alma1	ALNMAR (n/a)	(n/a) [c]	*Alnus glutinosa* Chr5 234,218-234,359	(CATT)3*	142	3e-69	100	glucan endo-1,3-beta-glucosidase 5 LOC133867598	Lance et al. 2009
alma11	ALNMAR (n/a)	KF724873	*Alnus glutinosa* Chr1 4,2675,090–42,675,369	(CT)2AT(CT)9…(CT)2	280	3e-113	91	Locus alma11, intergenic region	Lance et al. 2009
alng4	AGLUT (Y17713)	Y17713	*Alnus glutinosa* Chr2 30,520,551-30,520,678	T7(TTA)5*	128	8e-57	99	polcalcin Aln g 4 LOC133859370	Mingeot et al. 2010

[a] BETPLA *Betula platyphylla,* BETMAX *B. maximowicziana,* CORAVE *Corylus avellana,* ALNMAR *Alnus maritima, n/a not available*, AGLUT *Alnus glutinosa*.

[b] Gene Bank Number for SSR fragment amplified in *Alnus glutinosa* (Lepais et al. 2011; Drašnarová et al., 2014) and used for BLASTn similarity searches against *Alnus glutinosa* genome assembly dhAlnGlut1.
[c] Primer Forward (F: TCTGGCTCTGGGCTCTAAC) and Reverse (R: ATTCCTTCACTTCACCCGCT) were mapped against Alnus glutinosa genome assembly dhAlnGlut1 and 142bp long fragment were identified.
* SSR repeat units included in codifying sequence (exon).

Table S2. Genetic characterization of ten microsatellite loci in *A. cordata* and *A. glutinosa* species: for each locus total number of alleles (A), effective number of alleles (Ne), observed (H_o) and expected heterozygosity (H_E), polymorphic information content (PIC), null allele frequency estimated (F_{nul}) and unbiased estimate of Wright's fixation indices, within-population inbreeding coefficient f(F_{IS}), total-population inbreeding coefficient F (F_{IT}) and among-population genetic differentiation coefficient θ (F_{ST}) are shown.

Source	Locus	Size range (bp)	A	Ne	PIC	H_o	H_E	P-value [a]	F_{nul}	f (F_{IS}) [b]	F (F_{IT}) [b]	θ(F_{ST}) [b]	$F_{ST (null)}$
Alnus cordata (n = 417 [c])	A35	226-230	3	1.142	0.117	0.123	0.116	0.487	-0.0128	-0.025	-0.009	0.015	0.019
	A37	244-268	4	1.089	0.081	0.042	0.065	0.000***	0.2582	0.369***	0.442***	0.116***	0.150
	A38	100-118	3	1.484	0.280	0.340	0.318	0.441	-0.0196	-0.032	-0.034	0.001	0.000
	A2	142-145	2	1.635	0.323	0.345	0.374	0.005**	0.0737	0.083	0.140***	0.062***	0.061
	A22	162-180	8	4.280	0.775	0.810	0.746	0.744	-0.0155	-0.051	-0.027	0.022*	0.022
	AG10	201-240	11	2.256	0.472	0.584	0.542	0.999	-0.0175	-0.040	-0.030	0.010	0.012
	AG13	248-266	7	2.736	0.607	0.598	0.623	0.459	0.0558	0.083**	0.100**	0.018	0.018
	alma1	149	1	1.000	-	-	-	-	-	-	-	-	-
	alma11	195-358	7	1.853	0.425	0.452	0.446	0.896	0.0162	0.016	0.030	0.014	0.014
	alng4	126-147	11	1.655	0.374	0.304	0.367	0.000***	0.1368	0.208***	0.223***	0.019	0.025
	Total		57										
	Mean (SE)	-	5.700 (3.622)	1.913 (0.991)	0.384 (0.221)	0.400 (0.241)	0.400 (0.222)					0.031 (0.036)	0.035 (0.045)
Alnus glutinosa (n = 95)	A35	220-252	15	6.387	0.826	0.737	0.843	0.790	0.0666	0.100	0.135	0.038	0.037
	A37	238-256	8	3.163	0.656	0.505	0.684	0.000***	0.1218	0.248***	0.268***	0.026	0.025
	A38	106-132	7	2.441	0.567	0.537	0.590	0.189	0.0623	-0.028	0.111	0.136***	0.137
	A2	145-154	3	1.112	0.099	0.105	0.101	0.918	-0.0175	-0.056	-0.032	0.021	0.021

A22	160-176	7	3.014	0.628	0.642	0.668	0.982	0.0163	-0.005	0.051	0.056	0.057
AG10	204-236	13	4.039	0.730	0.600	0.752	0.038*	0.1041	0.104	0.220***	0.129***	0.121
AG13	248-272	11	5.191	0.786	0.774	0.807	0.058	0.0117	0.001	0.052	0.051	0.046
alma1	145-160	4	1.945	0.442	0.432	0.486	0.413	0.0516	-0.067	0.139	0.193***	0.193
alma11	341-370	16	7.896	0.861	0.853	0.873	0.923	0.0084	-0.034	0.037	0.069***	0.069
alng4	125-145	7	2.667	0.578	0.287	0.625	0.000***	0.3801	0.517***	0.547***	0.062	0.053
Total		91										
Mean (SE)		9.100 (4.458)	3.785 (2.115)	0.617 (0.223)	0.547 (0.228)	0.642 (0.224)					0.078 (0.056)	0.076 (0.056)

[a] P-value associated with likelihood ratio test (G-test) for departure from Hardy-Weinberg equilibrium: significant P-values are in bold (= p < 0.01).
[b] Level of significance of unbiased estimate of Wright's fixation indices were tested using a non-parametric approach described in Excoffier et al., (1992) with 1000 permutations: * = p < 0.05, **= p < 0.01, ***= p < 0.001
c Five individuals—SEV12, VIT1, SME15, SEV10, and SEV13—identified as multi-generation hybrids or backcrosses toward *A. cordata* through at least two methods, were excluded from the *A. cordata* dataset.

Table S3. Genetic diversity of *A. cordata* trees sampled in ten macro areas including five National (NP) and three Regional (RN) Parks: mean number of alleles per locus (A), effective number of alleles (Ne), allelic richness (Rs) and private allelic richness (PAr) standardized to 18 individuals from the original number of trees per population, observed (HO), expected (HE), and unbiased expected heterozygosity (UHE) and private alleles are shown.

Macro area	n	A	Ne	Rs	H_o	H_E	UH_E	Private alleles (Population)	
								Locus (N [a])	PAr
RP Partenio Mountains	21	29	1.953	2.850	0.324	0.356	0.364		0.000
RP Lattari Mountains	18	29	1.956	2.900	0.306	0.359	0.369	A37(256)†	0.100
RP Picentini Mountains	20	32	1.946	3.140	0.383	0.402	0.413		0.090
NP Cilento – Vallo di Diano	79	41	2.021	3.120	0.402	0.385	0.388	AG10(213)†, alma11(345)†, alng4(144)	0.210
NP Appennino Lucano Val d'Agri Lagonegrese	68	43	2.141	3.420	0.403	0.407	0.410	AG10(215)†, AG10(238), AG10(240), AG13(248)†, AG13(266)†, alng4(128)†	0.410
NP Pollino	98	39	1.928	3.090	0.339	0.360	0.362	alng4(146)	0.070
Paolano Appennines	45	34	1.958	3.080	0.331	0.358	0.362		0.020
NP Sila	24	33	1.899	3.160	0.366	0.380	0.388		0.070
RP Serre	24	26	1.799	2.550	0.345	0.345	0.352		0.000
NP Aspromonte	20	30	1.863	2.960	0.345	0.342	0.351		0.020

[a] Number of private alleles within the respective locus
† SSR allele shared with *A. glutinosa* species

Table S4. Results of the model selection assessing the effects of two geographic variables—latitude and elevation of sampled sites—and the mean estimated membership $Q_{2\_A.\ glutinosa}$ inferred by STRUCTURE in each *A. cordata* population on the allelic richness (Rs) and private allelic richness (Par) of *A. cordata* populations. Selected models with ΔAICc < 2 and with AICc value smaller than that of any simpler alternative, while accounting for nesting were reported. Selected models are shown in bold.

Response variable	Model	Variables retained	K	AICc	ΔAICc	Weight	Cum.Weight	LogLik
Rs	1	$Q_{2\_A.glutinosa}$ + Latitude	4	3.94	0.00	0.25	0.25	3.14
	**2**	**Latitude**	**3**	**4.8**	**0.86**	**0.16**	**0.41**	**1.23**
	**3**	**$Q_{2\_A.glutinosa}$**	**3**	**5.04**	**1.1**	**0.14**	**0.56**	**1.11**
PAr	**1**	**$Q_{2\_A.glutinosa}$**	**3**	**-93.44**	**0.00**	**0.46**	**0.46**	**50.35**
	2	$Q_{2\ A.glutinosa}$ + Latitude	4	-91.59	1.85	0.18	0.64	50.91

Table S5. For each selected model, coefficients of predictors and their statical significance are reported.

Response variable	Model	Predictor	β	SE	t-value	P
Rs	2	(Intercept)	-4.518	2.869	-1.574	0.13
		**Latitude**	**0.181**	**0.071**	**2.517**	**0.020 ***
	3	(Intercept)	2.598	0.066	39.359	<0.001***
		**$Q_{2\_A.glutinosa}$**	**0.266**	**0.108**	**2.458**	**0.023***
PAr	1	(Intercept)	0.003	0.007	0.431	0.671
		**$Q_{2\_A.glutinosa}$**	**0.098**	**0.012**	**7.732**	**<0.001*****

Table S6. Bottleneck analysis of 23 Italian alder populations sampled across Southern Italy using nine polymorphic nuclear microsatellite markers. Wilcoxon's signed-rank' test (Piry et al. 1999), shifted allele distribution analysis (Luikart et al. 1998) and the M-ratio test (Garza and Williamson 2001) for each Italian alder population are reported.

Population	Wilcoxon signed rank test [a]		L-shape [b]	M ratio value [c]
	Heterozygote deficiency	Heterozygote excess		
AVE	0.63281	0.41016	no deviation	0.77963
MOF	0.50000	0.54492	deviation	0.67778‡
BAI	0.45508	0.58984	no deviation	0.67568‡
LAU	0.90234	0.12500	no deviation	0.66426‡
SME	0.59375	0.46875	no deviation	0.58503‡
CAV	0.80859	0.23047	no deviation	0.76042
CHI	0.57813	0.47266	no deviation	0.71101
RAC	0.80859	0.23047	no deviation	0.74435
MAG	0.90234	0.12500	deviation	0.73194
SAS	0.72656	0.32031	no deviation	0.69271
ANZ	0.67969	0.37109	deviation	0.69203
SEV	0.37109	0.67969	no deviation	0.76042
NUP	0.58984	0.45508	no deviation	0.74938
BUO	0.47266	0.57813	deviation	0.72500
VIT	0.42188	0.62891	deviation	0.78333
ORS	0.36719	0.67383	no deviation	0.85185
SCA	0.19141	0.84375	no deviation	0.76736
CET	0.84961	0.17969	no deviation	0.73519
FIU	0.17969	0.84961	no deviation	0.79074
MAC	0.41016	0.63281	no deviation	0.72194
FOR	0.98145	0.02441*	deviation	0.81481
STA	0.00781**	1.00000	deviation	0.67444‡
SBR	0.45508	0.91016	no deviation	0.81111

[a] Significance of heterozygote excess and/or deficiency according to the Wilcoxon signed rank test under the Two-Phase Model of loci (TPM) for each walnut population: ** = $p < 0.01$, and * = $p < 0.05$.
[b] Deviation of allele frequency classes from a normal L-shaped.
[c] ‡ M ratio value smaller than the critical value 0.68.

Table S7. Results of the hierarchical analysis of molecular variance (AMOVA) considering ten macro areas. Genetic variation was partitioned into four levels: among macro areas, among populations within macro areas, among individuals within populations and within populations. Variance components, their percentages of the total variation, and F-statistics were estimated, and their significance was tested using 999 random permutations (* = $p < 0.05$, ** = $p < 0.01$, and *** = $p < 0.001$).

**Source of variation**	**df**	**Sum of squares**	**Variance component**	**% of variation**	***F*-statistic**
Among macro areas	9	39.445	0.017 Va	0.85	Φ_{CT} / F_{CT} = 0.0176*
Among populations within macro areas	13	37.591	0.030 Vb	1.55	Φ_{SC} / F_{SC} = 0.000***
Among individuals within populations	394	746.269	0.04716 Vc	2.49	Φ_{IS} / F_{IS} = 0.0078**
Within individuals	417	750.500	1.79976 Vd	95.07	Φ_{IT} / F_{IT} = 0.000***
Total	833	1573.805	1.89311	100.00	

Supplementary Figures

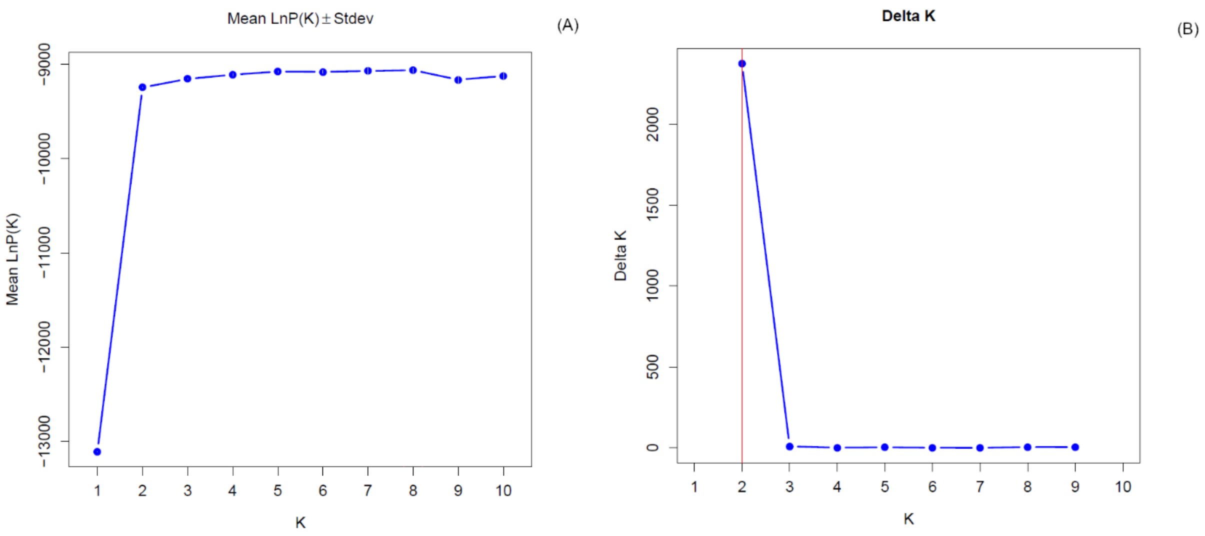

Figure S1. Hybridization between *A. cordata* and *A. glutinosa* considering the entire set of 518 alder tree collected across their native range in Italy. Inference of K, the most probable number of clusters based on microsatellite analysis of 518 alder samples using ten SSRs: Log-likelihood value of data L(K) as a function of K (A) and second order of change of the log-likelihood of the data (ΔK) as a function of K (B), calculated averaged over six replicates (Evanno et al. 2005).

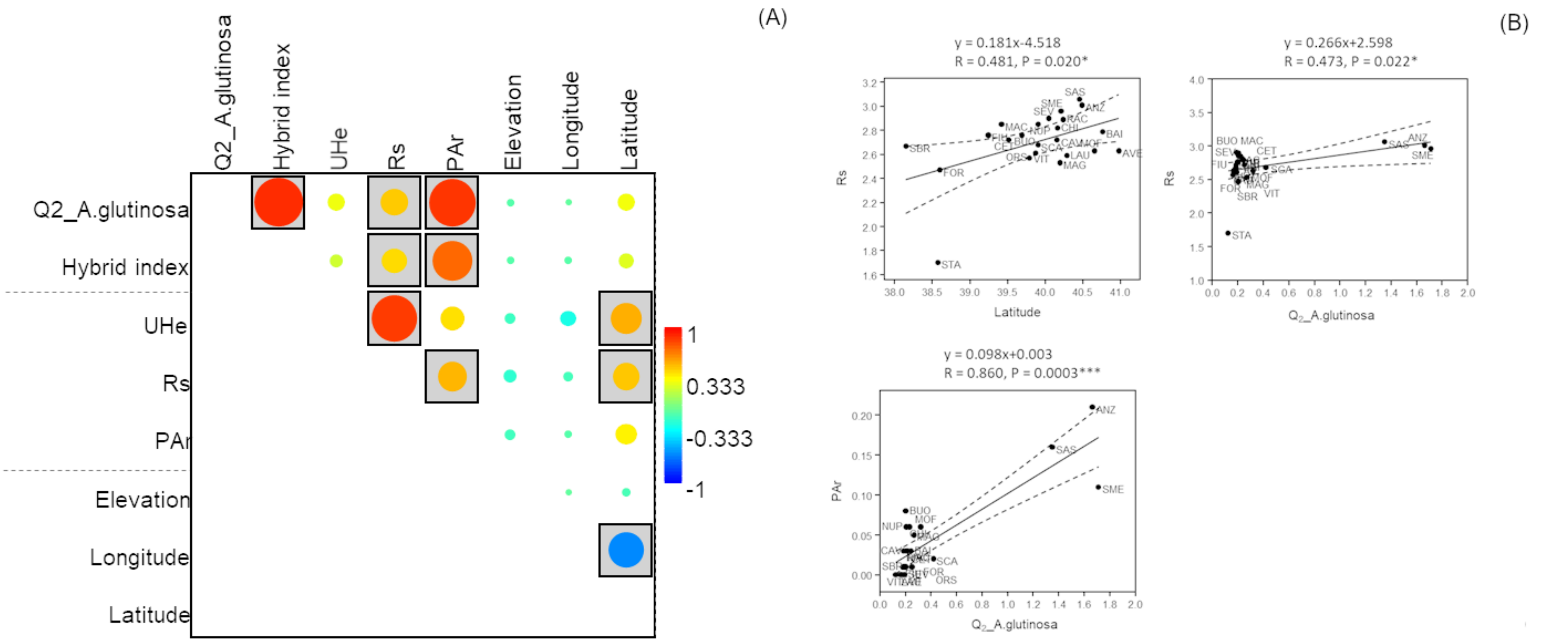

Figure S2. Heatmap of Pearson correlation between the unbiased expected heterozygosity (UH_E), allelic richness (Rs) and private allele richness (PAr) computed for each *A. cordata* population using nine polymorphic unlinked SSR markers and latitude, longitude, elevation of the sampling sites, mean estimated membership $Q2_{\_A.\ glutinosa}$ inferred in each population by STRUCTURE for K =2 and the mean hybrid index *per* population, measured as the proportion of alleles with *A. glutinosa* ancestry, computed by INTROGRESS. Significant Pearson correlation values for $P < 0.05$ were marked with a box (A). Individual Linear Modeling (LM) analysis of Rs and PAr against Latitude and $Q2_{\_A.\ glutinosa}$ is also displayed (B).

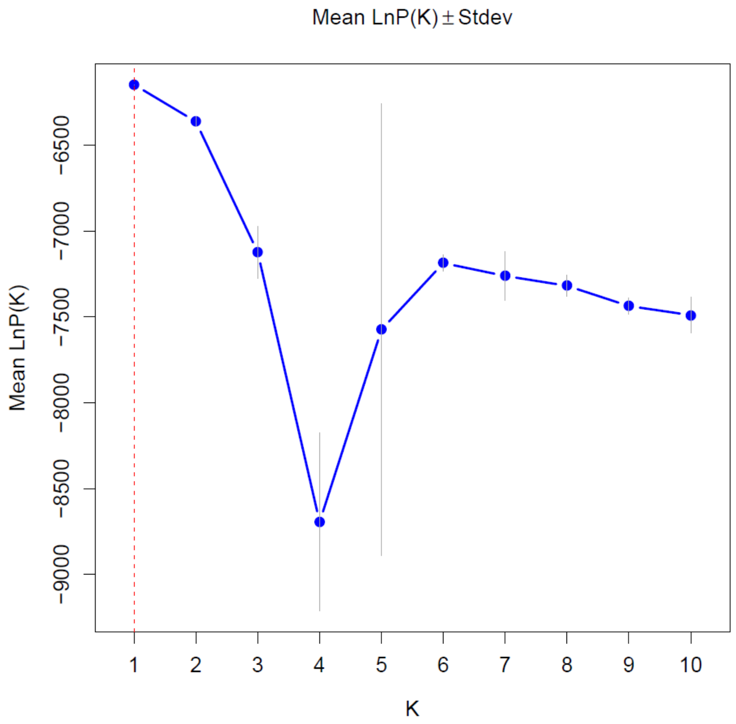

Figure S3. Inference of K, the most probable number of clusters, using STRUCTURE software (Pritchard et al. 2000), based on microsatellite analysis of all 417 Italian alder samples using nine polymorphic SSR markers. Log-likelihood value of data L(K) as a function of K averaged over six replicates was reported.

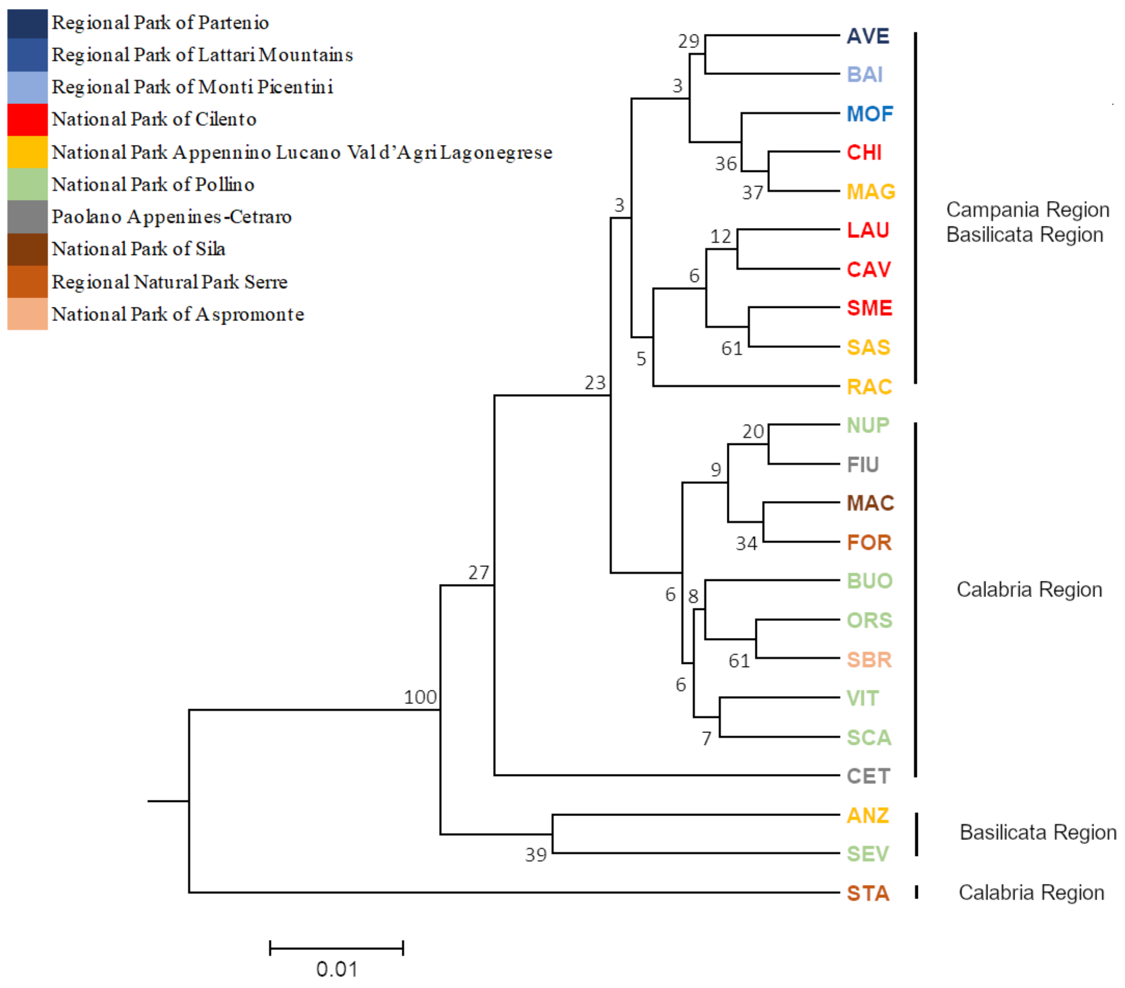

Figure S4. UPGMA-based tree of the 23 *A. cordata* populations collected in the Paolano Appenines and nine protected macro areas of Southern Italy based on Nei's genetic distance (1972). The number near each node represents the percentage of times when the node occurred among 1,000 bootstraps.

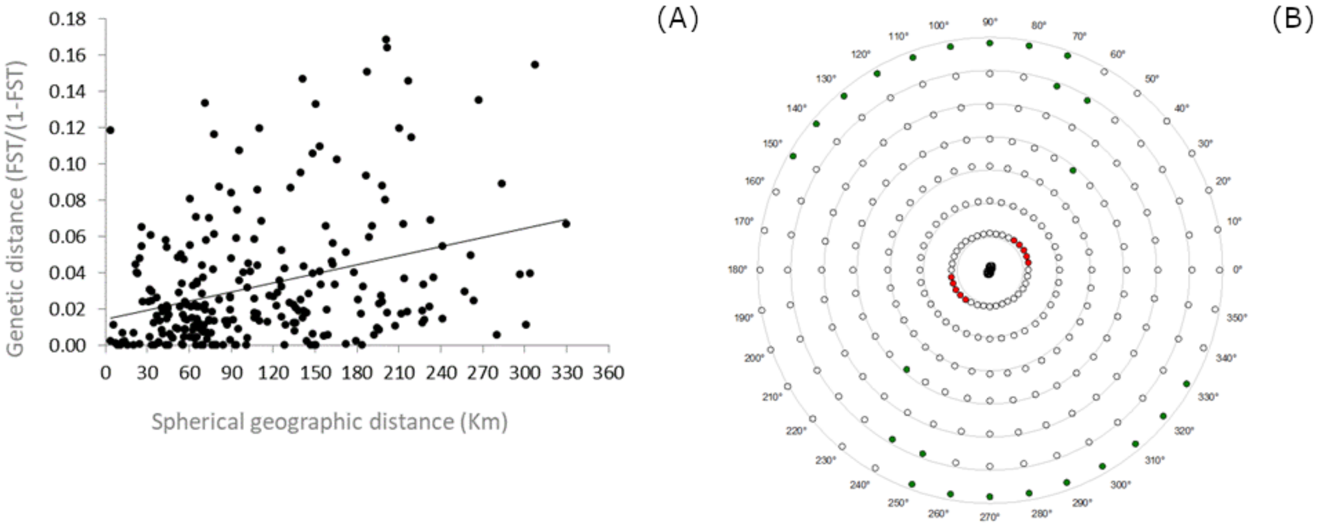

Figure S5. Correlation between genetic data of the *A. cordata* populations and geographic data. (A) Scatter plot of genetic (pairwise Slatkin's linearized distances [F_{ST} / (1- F_{ST})]) vs. geographical distances (spherical geographic distances in km) for pairwise population comparisons and (B) the subsequent Mantel Bearing correlogram within seven different geographic distance classes (upper limit of each annulus is 33.021, 53.217, 65.249, 76.886, 101.289, 134.370, 159.331 km). Red circles are positive autocorrelation ($P < 0.05$); green circles are significant negative autocorrelation ($P < 0.05$) and white cycles are nonsignificant.

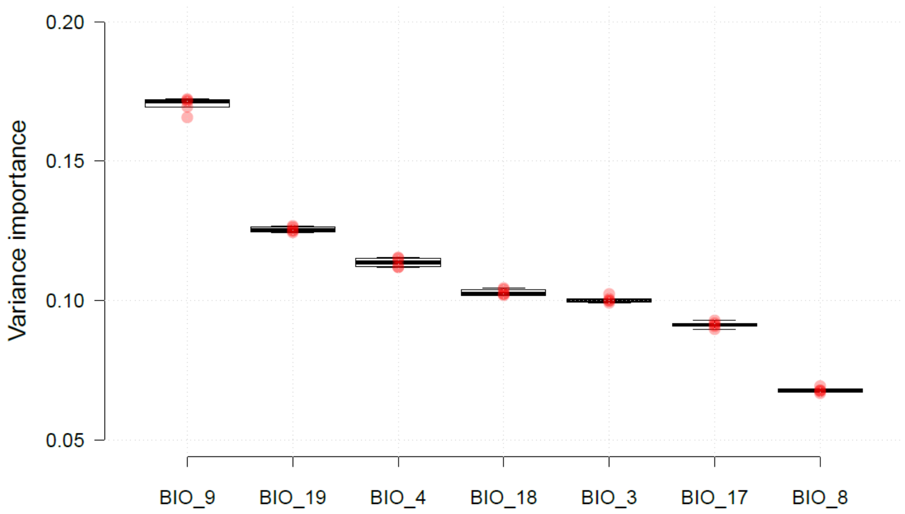

Figure S6. (A) Boxplots of the ensemble importance of all environmental variables [Isothermality (BIO3), Temperature Seasonality (BIO4), Mean Temperature of Wettest Quarter (BIO8), Mean Temperature of Driest Quarter (BIO9), Precipitation of Driest Quarter (BIO17), Precipitation of Warmest Quarter (BIO18) and Precipitation of Coldest Quarter (BIO19)] used to fit the SDMs of *A. cordata* species.

References

Chapuis, M.P., & Estoup, A. (2007). Microsatellite null alleles and estimation of population differentiation. *Molecular Biology and Evolution,* 24, 621–631. https://doi.org/10.1093/molbev/msl191

Drašnarová, A., Krak, K., Vít, P., Doudová, J., Douda, J., Hadincová, V., ... & Mandák, B. (2014). Cross-amplification and multiplexing of SSR markers for Alnus glutinosa and A. incana. Tree Genetics & Genomes, 10, 865-873.

Evanno, G., Regnaut, S., & Goudet, J. (2005) Detecting the number of clusters of individuals using the software STRUCTURE: a simulation study. *Molecular Ecology*, 14, 2611–2620. https://doi.org/10.1111/j.1365-294X.2005.02553.x

Excoffier, L., Smouse, P. E., & Quattro, J. M. (1992). Analysis of molecular variance inferred from metric distances among DNA haplotypes: application to human mitochondrial DNA restriction data. Genetics, 131(2), 479-491.

Garza, J.C., & Williamson, E.G. (2001) Detection of reduction in population size using data from microsatellite loci. *Molecular Ecology*, 10, 305–318. https://doi.org/10.1046/j.1365-294X.2001.01190.x

Gürcan, K., Mehlenbacher, S.A., Botta, R., Boccacci, P. (2010). Development, characterization, segregation, and mapping of microsatellite markers for European hazelnut (Corylus avellana L.) from enriched genomic libraries and usefulness in genetic diversity studies. *Tree Genetics & Genomes* 6, 513–531. https://doi.org/10.1007/s11295-010-0269-y

Lance, S. L., Jones, K. L., Hagen, C., Glenn, T. C., Jones, J. M., & Gibson, J. P. (2009). Development and characterization of nineteen polymorphic microsatellite loci from seaside alder, Alnus maritima. *Conservation genetics*, 10, 1907-1910. https://doi.org/10.1007/s10592-009-9851-y

Lepais, O., & Bacles, C. F. E. (2011). De novo discovery and multiplexed amplification of microsatellite markers for black alder (Alnus glutinosa) and related species using SSR-enriched shotgun pyrosequencing. *Journal of Heredity*, 102(5), 627-632. https://doi.org/10.1093/jhered/esr062

Luikart, G., Allendorf, F.W., Cornuet, J.M., & Sherwin, W.B. (1998) Distortion of allele frequency distributions provides a test for recent population bottlenecks. *Journal of Heredity*, 89, 238–247. https://doi.org/10.1093/jhered/89.3.23

Mingeot, D., Baleux, R., & Watillon, B. (2010). Characterization of microsatellite markers for black alder (Alnus glutinosa [L.] Gaertn). *Conservation Genetics Resources*, 2, 269-271.

Nei, M (1972) Genetic distance between populations. *The American Naturalist*, 106, 283–292.

Piry, S., Luikart, G., & Cornuet, J.M. (1999) BOTTLENECK: a computer program for detecting recent reductions in effective population size from allele frequency data. *Journal of Heredity*, 90, 502–503. https://doi.org/10.1093/jhered/90.4.502

Pollegioni, P., Woeste, K., Chiocchini, F., Olimpieri, I., Tortolano, V., Clark J.,…& Malvolti M.E. (2014). Landscape genetics of Persian walnut (Juglans regia L.) across its Asian range. *Tree Genetics and Genomes,* 10:1027–1043. https://doi.org/10.1007/s11295-014-0740-2

Pritchard, J.K., Stephens, M., &Donnelly, P. (2000) Inference of population structure using multilocus genotype data. *Genetics*, 155, 945–959. https://doi.org/10.1093/genetics/155.2.945

Rousset, F., (2008). Genepop'007: a complete reimplementation of the Genepop software for Windows and Linux. *Molecular Ecology Resources*, 8, 103-106. https://doi.org/10.1111/j.1471-8286.2007.01931.x

Tsuda, Y., Ueno, S., Ide, Y., & Tsumura, Y (2009a). Development of 14 EST-SSRs for Betula maximowicziana and their applicability to related species. *Conservation Genetics*, 10, 661–664. https://doi.org/10.1007/s10592-008-9608-z

Tsuda, Y., Ueno, S., Ranta, J., Salminen, K., Ide, Y., Shinohara, K., & Tsumura, Y (2009b). Development of 11 EST-SSRs for Japanese white birch, Betula platyphylla var. japonica and their

transferability to related species. *Conservation Genetics*, 10, 1385–1388. https://doi.org/10.1007/s10592-008-9701-3

Wu, B., Lian, C., & Hogetsu, T. (2002), Development of microsatellite markers in white birch (Betula platyphylla var. japonica). *Molecular Ecology Notes*, 2, 413-415. https://doi.org/10.1046/j.1471-8286.2002.00260.x
